# Global rice methane emissions tripled over six decades amid declining emission intensity

**DOI:** 10.1093/nsr/nwag555

**Published:** 2026-08-29

**Authors:** Qiwen Hu, Tingting Li, Hanzhi Xie, Yao Huang, Josep G Canadell, Jingxian Li, Pete Smith, Wenping Yuan, Wen Zhang, Jianwen Zou, Jing Liu, Jiawen Zhang, Zhangcai Qin, Shilong Piao

**Affiliations:** School of Atmospheric Sciences, Guangdong Province Data Center of Terrestrial and Marine Ecosystems Carbon Cycle, Sun Yat-sen University, Zhuhai 519000, China; State Key Laboratory of Atmospheric Environment and Extreme Meteorology, Institute of Atmospheric Physics, Chinese Academy of Sciences, Beijing 100029, China; Southern Marine Science and Engineering Guangdong Laboratory (Zhuhai), Zhuhai 519000, China; School of Atmospheric Sciences, Guangdong Province Data Center of Terrestrial and Marine Ecosystems Carbon Cycle, Sun Yat-sen University, Zhuhai 519000, China; State Key Laboratory of Atmospheric Environment and Extreme Meteorology, Institute of Atmospheric Physics, Chinese Academy of Sciences, Beijing 100029, China; State Key Laboratory of Vegetation and Environmental Change, Institute of Botany, Chinese Academy of Sciences, Beijing 100093, China; Global Carbon Project, CSIRO Environment, Canberra, ACT 2601, Australia; School of Atmospheric Sciences, Guangdong Province Data Center of Terrestrial and Marine Ecosystems Carbon Cycle, Sun Yat-sen University, Zhuhai 519000, China; Institute of Biological and Environmental Sciences, University of Aberdeen, Aberdeen AB24 3UU, UK; Institute of Carbon Neutrality, College of Urban and Environmental Sciences, Peking University, Beijing 100871, China; State Key Laboratory of Atmospheric Environment and Extreme Meteorology, Institute of Atmospheric Physics, Chinese Academy of Sciences, Beijing 100029, China; College of Resources and Environmental Sciences, Nanjing Agricultural University, Nanjing 210095, China; School of Atmospheric Sciences, Guangdong Province Data Center of Terrestrial and Marine Ecosystems Carbon Cycle, Sun Yat-sen University, Zhuhai 519000, China; School of Atmospheric Sciences, Guangdong Province Data Center of Terrestrial and Marine Ecosystems Carbon Cycle, Sun Yat-sen University, Zhuhai 519000, China; School of Atmospheric Sciences, Guangdong Province Data Center of Terrestrial and Marine Ecosystems Carbon Cycle, Sun Yat-sen University, Zhuhai 519000, China; Southern Marine Science and Engineering Guangdong Laboratory (Zhuhai), Zhuhai 519000, China; Institute of Carbon Neutrality, College of Urban and Environmental Sciences, Peking University, Beijing 100871, China

**Keywords:** rice paddies, methane budget, emission intensity, process-based modeling, machine learning

## Abstract

Rice paddies are among the largest anthropogenic sources of methane (CH_4_), yet substantial uncertainties persist in the long-term magnitude and spatiotemporal dynamics of the emissions. Here, we show a spatially explicit global estimate of rice CH_4_ emissions from 1961 to 2020, leveraging a new high-resolution dataset and two Tier 3 modeling approaches (process-based and machine-learning-based). We find that global emissions have more than tripled over the past six decades, reaching a record high of 38.8 Tg CH_4_ yr^−1^ in 2020 based on the mean of the two Tier 3 estimates, with both the rate and magnitude substantially higher than those of conventional emission-factor-based inventories. Traditional rice-producing regions (e.g. Asia) dominate the increase due to expanded cultivation and intensified organic inputs, while Africa emerged as a rapidly growing source. Beyond trends in absolute emissions, the CH_4_ emission intensity (per unit of rice production) declined across 70% of the global paddy area, mainly reflecting yield-driven efficiency gains. Notably, 44% of global rice production in the 2010s occurred in countries with below-average emission intensity, with China being the largest contributor. By resolving long-term spatial heterogeneity with new data and models, this study provides a robust reassessment of the global rice methane budget and highlights pathways to reconcile food production with climate mitigation.

## Teaser

Global rice methane emissions have more than tripled since 1961, yet emission intensity has declined across much of the world, highlighting divergent regional trajectories and untapped mitigation potential.

## INTRODUCTION

Methane (CH_4_), the second-largest contributor to anthropogenic warming after carbon dioxide, has contributed more than 0.5°C to the global mean surface temperature increase to date [1]. Owing to its high global warming potential (84 times higher than carbon dioxide over a 20-year time horizon) and relatively short atmospheric lifetime (9–12 years), CH_4_ mitigation offers an effective pathway for near-term climate benefits [1]. Among anthropogenic CH_4_ sources, flooded rice paddies are one of the largest individual sources, contributing 7%–10% of the global anthropogenic share [2,3]. Given that rice is a staple food for over half of the world’s population [4], mitigating rice-related CH_4_ emissions presents a fundamental challenge for reconciling climate mitigation with food security.

Estimates of rice CH_4_ emissions remain highly uncertain across existing inventories. Depending on assumptions regarding emission factors (EFs) and activity data, global emissions in 2020 range from 20 to 37 Tg CH_4_ yr^−1^, a nearly two-fold difference [4–8]. This uncertainty is reflected in successive IPCC assessments, where estimated CH_4_ emissions from rice paddies during 2000–2009 were revised from 36 (33–40) Tg CH_4_ yr^−1^ in the Fifth Assessment Report (AR5) to 29 (25–37) Tg CH_4_ yr^−1^ in AR6 [1,2]. While part of this discrepancy reflects the complexity of CH_4_

production under anaerobic conditions and its sensitivity to organic inputs, water management, temperature, and soil properties [9–12], a more fundamental limitation lies in the widespread reliance on EF approaches.

Within the IPCC framework, rice CH_4_ emissions are commonly estimated using a tiered approach. Tier 1 and Tier 2 methods rely on EFs applied to aggregated activity data, with Tier 2 incorporating country-specific parameters or management information. These approaches enable broad global coverage but typically assume spatially and temporally uniform emission rates within large climate zones or management categories [13–15]. Such assumptions limit their ability to capture subnational variability and long-term trends in environmental and agronomic conditions. For instance, warming or increased organic amendments can substantially affect CH_4_ production but remain unaccounted for in static EF frameworks [16].

To overcome these limitations, Tier 3 approaches, including data-driven and process-based modeling [14,17–19], have increasingly been explored and offer opportunities to refine estimates. Data-driven methods, including machine learning, can capture regional patterns when sufficient field data exist, but often lack mechanistic interpretability and struggle in data-sparse regions [16]. Process-based models simulate microbial processes governing CH_4_ dynamics and offer a balance between physical realism and global scalability [18,19]. However, their application at the global scale has been challenged by the historical lack of spatially and temporally resolved input datasets.

Meanwhile, rice production has expanded dramatically, driven by population growth, economic development, and dietary changes, especially in developing countries [20–22]. Global rice consumption increased from 216 Mt in 1961 to over 770 Mt in 2022 [4], underscoring the growing importance of rice systems in the context of both food and climate. While regional-scale studies have provided valuable insights [23–26], significant gaps remain in quantifying long-term global trends in rice CH_4_ emissions and identifying their underlying environmental and management drivers. Moreover, because rice is a staple food crop, evaluating the climate impact of rice systems requires not only total emissions but also emission intensity, defined as CH_4_ emissions per unit of rice production. This metric links methane mitigation to food security by distinguishing production-driven growth in emissions from changes in emission efficiency.

To address these gaps, we developed a global, high-resolution assessment of CH_4_ emissions from rice paddies from 1961 to 2020 using three structurally distinct yet harmonized approaches that span the main methodological frameworks to estimate rice CH_4_ emissions. Two Tier 3 approaches, a process-based model (CH4MOD) and a machine-learning model (CH4-ML) trained on field observations, explicitly account for spatial and temporal variations in climate, water management, and organic inputs. Both are driven by a novel 5-arcmin global dataset integrating rice area, phenology, temperature, soil characteristics, and management practices. For comparison, we also implemented an improved Tier 2 emission-factor-based method (CH4-EF), with gridded activity data and calibrated scaling factors. By integrating multiple estimation approaches, high-resolution harmonized datasets, and multiple lines of evaluation, this framework helps constrain method-dependent uncertainty and provides a more robust evidence base for reassessing long-term trends in global rice CH_4_ emissions. It also provides a quantitative basis for disentangling the roles of environmental change, agricultural intensification, and production efficiency in shaping emission trajectories.

## RESULTS

### Rapid increase in global rice CH_4_ emissions

Using the three structurally distinct approaches, a process-based model (CH4MOD), a machine-learning approach (CH4-ML), and an improved EF-based approach (CH4-EF), we consistently identify a pronounced increase in global rice CH_4_ emissions since 1961 (Fig. 1a). CH4MOD estimates that CH_4_ emissions increased from 10.1 Tg CH_4_ yr^−1^ in 1961 to 39.5 Tg CH_4_ yr^−1^ in 2020, with an average annual increase of 0.49 Tg CH_4_ yr^−1^. CH4-ML reveals a similar upward trajectory, rising from 13.9 to 38.1 Tg CH_4_ yr^−1^, corresponding to a growth rate of 0.40 Tg CH_4_ yr^−1^. The CH4-EF approach also indicates a substantial rise, from 18 to 33 Tg CH_4_ yr^−1^ over the same period (0.25 Tg CH_4_ yr^−1^). Collectively, these results demonstrate that global rice CH_4_ emissions have at least doubled and more likely tripled over the past six decades.

**Figure 1.**
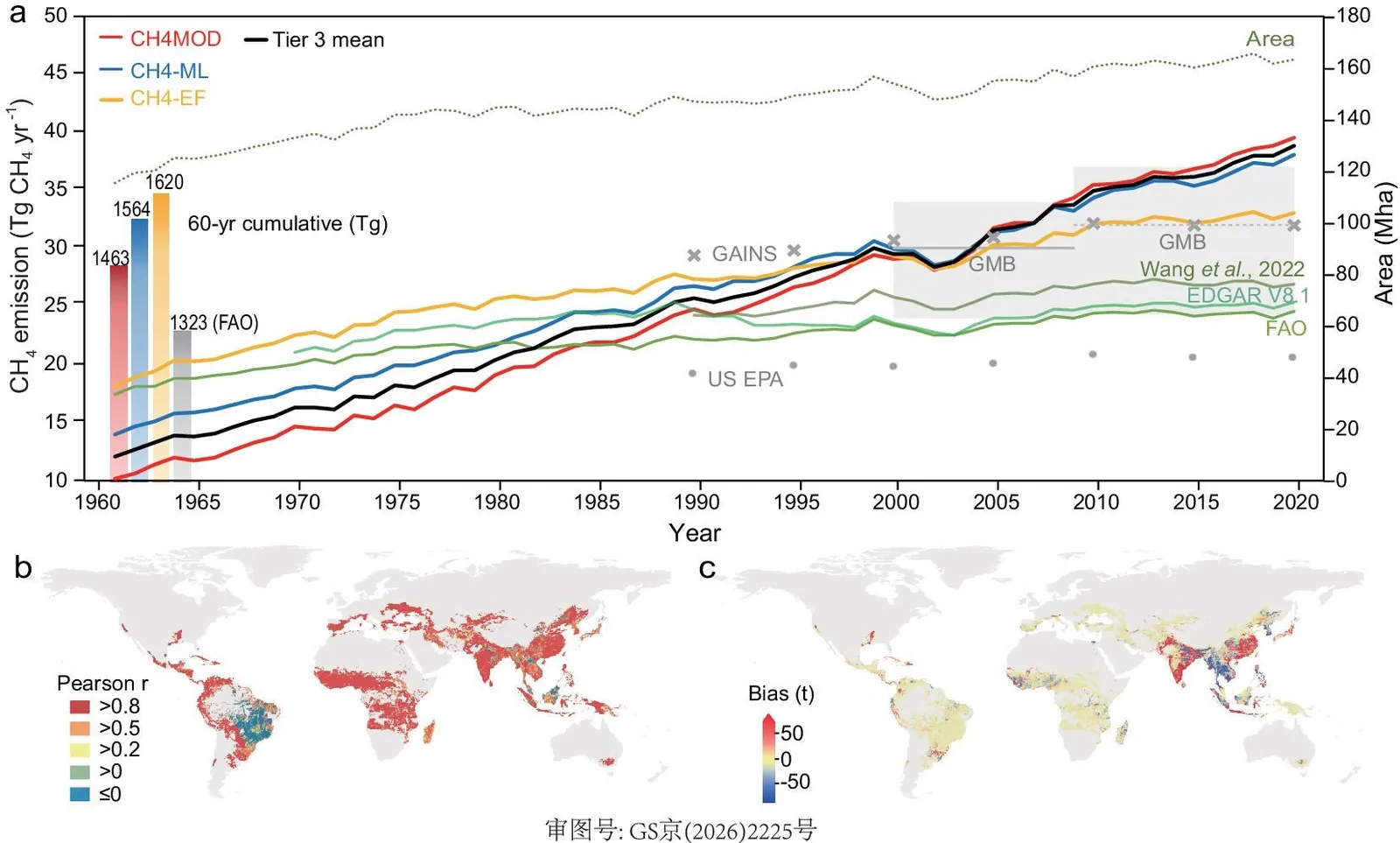
Global CH_4_ emissions from rice paddies. (a) Global CH_4_ emissions from 1961 to 2020 estimated in this and other specified studies. Estimates from the process-based model (CH4MOD), machine learning (CH4-ML), their Tier 3 mean, and the improved IPCC emission-factor-based approach (CH4-EF) are shown. The Tier 3 mean is defined as the average of CH4MOD and CH4-ML. The shaded area with CH4-EF represents the range of estimates based on the 2006 and 2019 IPCC guidelines. Cumulative CH_4_ emissions over 1961–2020 from CH4MOD, CH4-ML, CH4-EF, and FAO are also shown as bar charts. See Methods for modeling description. Other studies with specific estimates include Global Methane Budget [3] (GMB, the shaded areas represent the range), FAO [4], EDGAR [5], US EPA [6], GAINS [7], Wang *et al*. [8]. (b) Grid-level Pearson correlation coefficients (*r*) between CH4MOD and CH4-ML CH_4_ emission time series over 1961–2020. (c) Spatial distribution of differences between the Tier 3 mean and CH4-EF estimates during 2010s.

In sharp contrast, conventional Tier 1 inventories, including FAO, EDGAR, GAINS, US EPA, and Wang *et al*. (2023) [4–8], report much slower growth rates, typically ranging from 0.04 to 0.12 Tg CH_4_ yr^−1^ (Fig. 1a). These inventories largely attribute changes in emissions to variations in harvested area, reflecting their reliance on static EFs and aggregated regional activity data, although EFs may vary by country or continent (Table S1). Such approaches do not explicitly represent long-term changes in temperature, rice productivity, or organic substrate supply, and therefore fail to capture intensification-driven increases in CH_4_ production. Among these inventories, the FAO dataset provides the most consistent temporal record and is therefore used as a reference for comparison. Over the past 60 years, FAO estimates have increased by 1.4 times, with annual emissions in 2020 remaining 35%–38% lower than those from Tier 3 estimates of CH4MOD and CH4-ML. These differences highlight the tendency of EF-based inventories to underestimate long-term emission growth by neglecting changes in productivity, organic substrate availability, and temperature.

Our improved Tier 2 approach (CH4-EF), which utilizes grid-scale harvested area, rice yield, organic amendment input, and spatially explicit water regime information (Table S1), enhances spatiotemporal representativeness and substantially reduces the underestimation observed in Tier 1 inventories. Its cumulative emissions over 1961–2020 (1620 Tg) closely match those of CH4MOD and CH4-ML. Nevertheless, its annual emission in 2020 remains 13%–16% lower than the two Tier 3 estimates, indicating that even an enhanced emission-factor approach may incompletely represent biogeochemical feedbacks associated with warming and yield-driven increases in methanogenic substrates. Notably, the three approaches converge around the early 2000s, likely reflecting the influence of extensive field observations concentrated in the 1990s and 2000s, which provided stronger empirical constraints on EFs.

The two Tier 3 estimates, CH4MOD and CH4-ML, exhibit highly consistent spatiotemporal variability. Grid-level time series from 1961 to 2020 exhibit strong temporal correlations across major rice-growing regions (Fig. 1b, *r* predominantly > 0.8), and their 2010s mean spatial patterns are similarly aligned (global *r* = 0.89), indicating that the major spatial patterns and long-term trends in rice CH_4_ emissions are robust.

However, the improved Tier 2 approach of CH4-EF is systematically different from the Tier 3 approaches in terms of spatial CH_4_ emissions (Fig. 1c). For 2010s, Tier 3 estimates are generally higher than CH4-EF in major high-emission rice systems, including densely cultivated regions of China, India, and Indonesia. In contrast, the CH4-EF tends to smooth subregional variability and introduces region-specific biases. In major rice-producing areas of Southeast Asia, CH4-EF consistently and systematically overestimates emissions by 30%–80%. In Africa, CH4-EF also shows systematic overestimation, largely because the global-average EF applied to this region is higher than the emission level indicated by Tier 3 estimates; this bias has been amplified in recent decades as rice area has expanded rapidly. These coherent spatial biases reflect the inherent tendency of emission-factor approaches to smooth subregional variability and obscure differences among cropping systems and management practices. The overall agreement of the two Tier 3 approaches, as opposed to the improved Tier 2 method, suggests that conventional inventories have likely underestimated the magnitude and growth rate of rice CH_4_ emissions in recent decades.

### Regional contribution to methane emissions

Emissions estimated from the Tier 3 mean (average of CH4MOD and CH4-ML) exhibit pronounced spatial heterogeneity in their long-term evolution. Grid-scale relative emission changes over time range from −0.5 to over 300 times (Fig. 2a). Large relative increases occur mainly in emerging rice-growing regions, particularly in sub-Saharan Africa and parts of South America, where historical emission levels were low and rice cultivation expanded rapidly. In contrast, major emitting countries such as China and India show moderate relative growth. Additionally, some countries, such as Australia and Japan, experience declining emissions over this period due to reductions in crop area.

**Figure 2.**
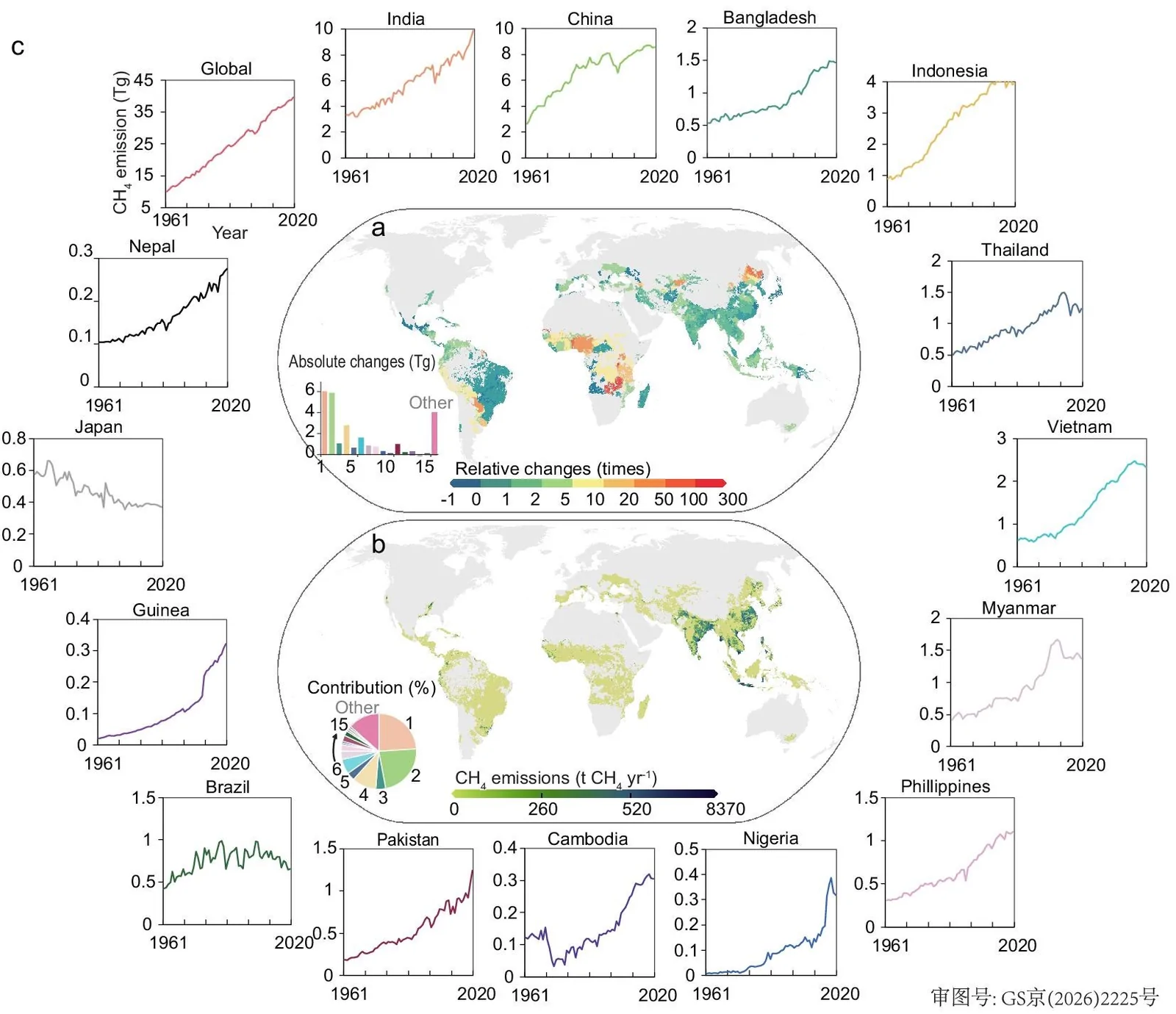
Regional CH_4_ emissions. (a) Spatial distribution of changes in annual CH_4_ emissions from rice paddies in 2010s relative to 1960s, with histograms showing national-level absolute changes; (b) spatial distribution of CH_4_ emissions in 2010s, with pie charts showing national contributions to the global total; (c) time series of CH_4_ emissions in 15 major rice-producing countries from 1961 to 2020, with arrows indicating relative changes over 60 years. The values are estimated by the average of CH4MOD and CH4-ML. The colors in histograms (a), pie charts (b), and plots (c) indicate the countries shown in (c). The 15 major rice-producing countries include (1) India, (2) China, (3) Bangladesh, (4) Indonesia, (5) Thailand, (6) Vietnam, (7) Myanmar, (8) Philippines, (9) Nigeria, (10) Cambodia, (11) Pakistan, (12) Brazil, (13) Guinea, (14) Japan, and (15) Nepal.

By the 2010s, global rice CH_4_ emission hotspots remain largely concentrated in key rice-production regions across Asia, where intensive cultivation systems dominate (Fig. 2b). India (24%), China (23%), Indonesia (10.4%), and Vietnam (6.4%) together account for ∼63% of the global rice CH_4_ emissions during the 2010s. Among the top 15 rice-producing countries that currently account for 89% of the global harvested area, the share of emissions declines from over 90% in 1961 to ∼86% in 2020, reflecting a growing contribution from emerging rice-growing regions outside the traditional production centers.

Long-term emission trajectories of these top 15 producers reveal pronounced regional heterogeneity (Fig. 2c). Global emissions increase by 26.78 Tg from 1961 to 2020, with India contributing the largest absolute increase (6.56 Tg, 25% of the total), followed by China (23%), Indonesia (11%), and Vietnam (6%). Bangladesh, Myanmar, and Pakistan each account for ∼4% of the global increase (Fig. 2a and Table S2). Despite their comparable cumulative contributions, China and India exhibit increasingly divergent emission trajectories in recent two decades. Emissions in India continue to rise steadily throughout the study period, whereas emissions in China increased rapidly in earlier decades but show a marked slowdown from the 1990s onward, resulting in a relative stabilization in recent years. As a result, the two largest rice-producing countries follow distinct pathways, underscoring substantial heterogeneity in national contributions to global rice methane trends.

Other major producers also display diverse patterns. Indonesia, Thailand, and the Philippines also exhibit sustained growth, with emissions increasing by factors of 4.3, 2.5, and 3.7, respectively. In contrast, Bangladesh, Vietnam, Myanmar, and Cambodia experience rapid increases in emissions starting in the 1980s. Some emerging rice-growing countries, including Nigeria and Guinea, show large proportional increases from low initial emission levels, while their absolute contributions to the global increase remain comparatively small (Table S2). Brazil shows only a modest increase of 1.5 times, whereas Japan’s emissions decline to 65% of their 1961 levels.

### Drivers of rising emissions

The rapid increase in global rice CH_4_ emissions since 1961 is jointly driven by the expansion of rice paddy areas and the increase in emissions per unit area (Fig. 3). Global rice harvested area increases by ∼42% over the study period (Fig. S1), although both the magnitude and spatial pattern of expansion are highly uneven (Fig. 3a). In traditional rice-producing regions of Asia, rice paddy area expands at a moderate pace, with more notable increases in Pakistan, Thailand, and Vietnam (1.6–2 times). In contrast, Sub-Saharan Africa and parts of South America show large proportional expansions in rice cultivation from low initial cultivated areas. Conversely, countries like Brazil and Japan have experienced a nearly 50% reduction in rice paddy area.

**Figure 3.**
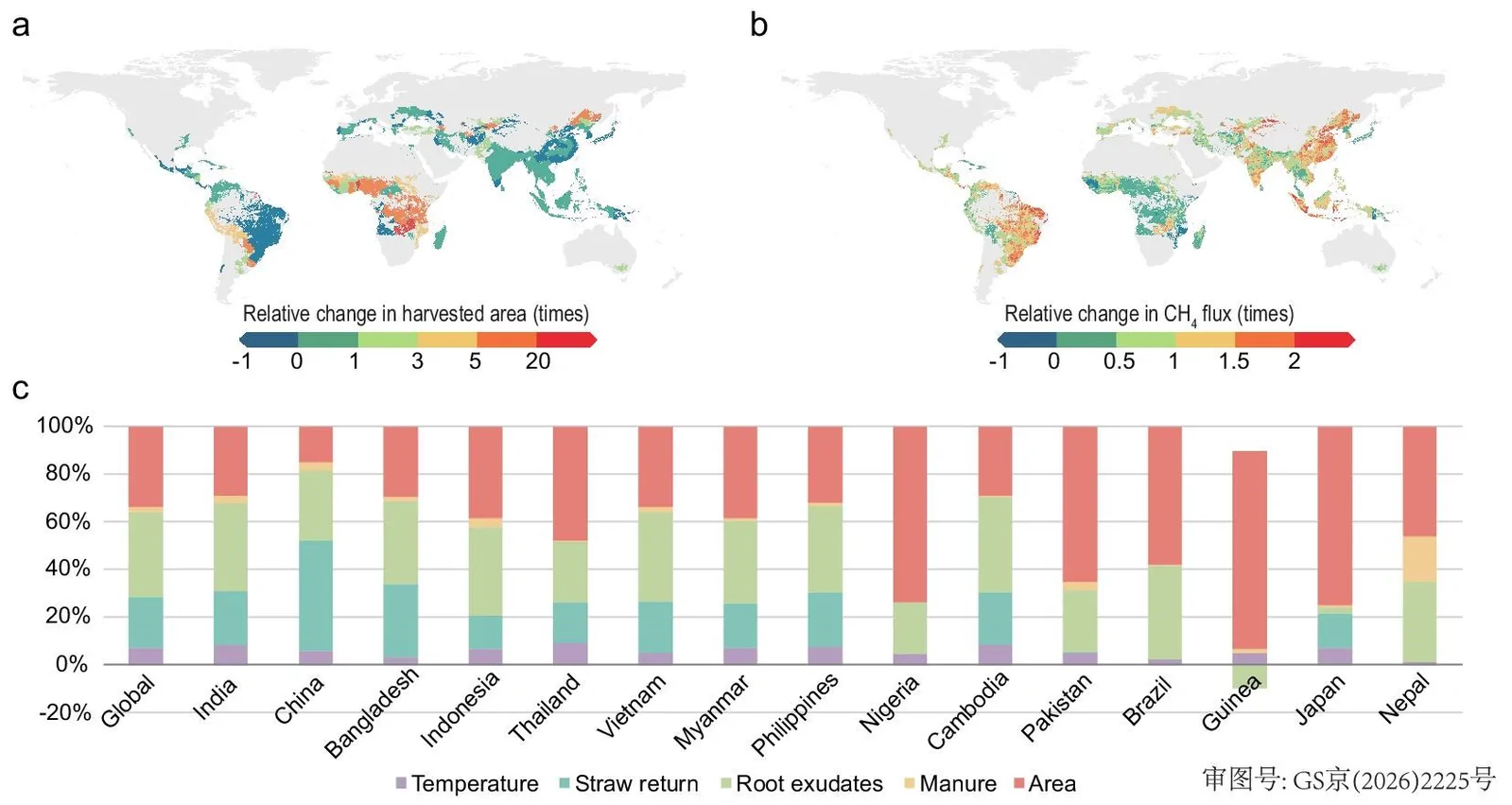
Global rice expansion and CH_4_ flux increase. (a) Change of rice harvested area in the 2010s relative to the 1960s and (b) change of CH_4_ flux from rice paddies in the 2010s relative to the 1960s. The values are averaged over CH4MOD and CH4-ML. (c) contribution of factors to the change in CH4MOD estimated CH_4_ emissions from rice paddies globally and in the top 15 rice-producing countries from 1961 to 2020.

The spatial heterogeneity in CH_4_ flux per unit area estimated by the average of CH4MOD and CH4-ML further amplifies the spatial divergence in total emissions (Fig. 3b). Notably, emission fluxes increase significantly in coastal regions of Brazil, while traditional rice-producing countries in Asia exhibit moderate increases, with East and South Asia showing greater growth than Southeast Asia. In contrast, changes in emission flux remain relatively small in Africa despite the rapid expansion of rice cultivation area.

To quantify the apparent contribution of individual drivers, we performed a fixed-driver decomposition using CH4MOD (Fig. 3c). Globally, increased root exudates resulting from yield increases are the largest contributor to the increase in emissions (36%), followed by expansion of rice area (33%) and increased straw incorporation (21%). Temperature rise explains ∼7% of the global increase, while changes in manure application contribute only ∼3%. These results indicate that yield-driven increases in methanogenic substrate supply, together with expansion of rice cultivation, dominate the long-term growth of rice CH_4_ emissions.

National-level decomposition reveals substantial heterogeneity in driver importance. Area expansion is the primary driver in African countries, Thailand, and Pakistan, whereas it contributes little to emission growth in China. In Brazil and Japan, reductions in cultivated area offset part of the potential emission increase. Temperature correlates positively with CH_4_ emissions but plays a relatively small role compared to the other drivers, and its impact is more uniform across countries (4%–8%). In CH4MOD, temperature affects emissions through direct stimulation of methanogenesis, while its effect on rice yield is not represented, as yield is externally prescribed. Consequently, indirect climate effects on productivity are not attributed to the temperature term in this decomposition but are embedded in the observed yield-driven substrate changes.

Increased root exudates contribute strongly in Cambodia, Vietnam, India, the Philippines, and Bangladesh, where rapid yield growth but relatively low straw incorporation rates make root exudates the primary source of methanogenic carbon. In Guinea, however, declining rice yields have reduced root exudates, partially offsetting the emission increase from area expansion. In contrast, straw incorporation is a major contributor in China, where steady yield increases and widespread residue return push its contribution to as high as 47%. Manure application plays a minor role in most countries, with the notable exception of Nepal, where its contribution has increased substantially. Overall, root exudates and straw incorporation serve as key substrates for CH_4_ production influenced by rice yields, making them crucial contributors to emission growth [18,27,28].

### CH_4_ emissions and rice yield

Global rice production has increased over the past six decades and so have CH_4_ emissions. The balance between emissions and rice yield varies largely by region. From 1961 to 2020, global mean rice yield increased 2.6 times (from 188 to 481 g m^−2^), while CH_4_ fluxes per unit area doubled (from 13 to 26 g CH_4_ m^−2^) (Fig. 4a). Over the same period, global emission intensity, defined as total emissions per unit of rice production, declined from 50 to 45 g CH_4_/kg rice (Fig. 4b). This decline mainly reflects yield growth outpacing the increase in CH_4_ emissions per unit area, indicating a production efficiency gain rather than direct evidence of active methane mitigation.

**Figure 4.**
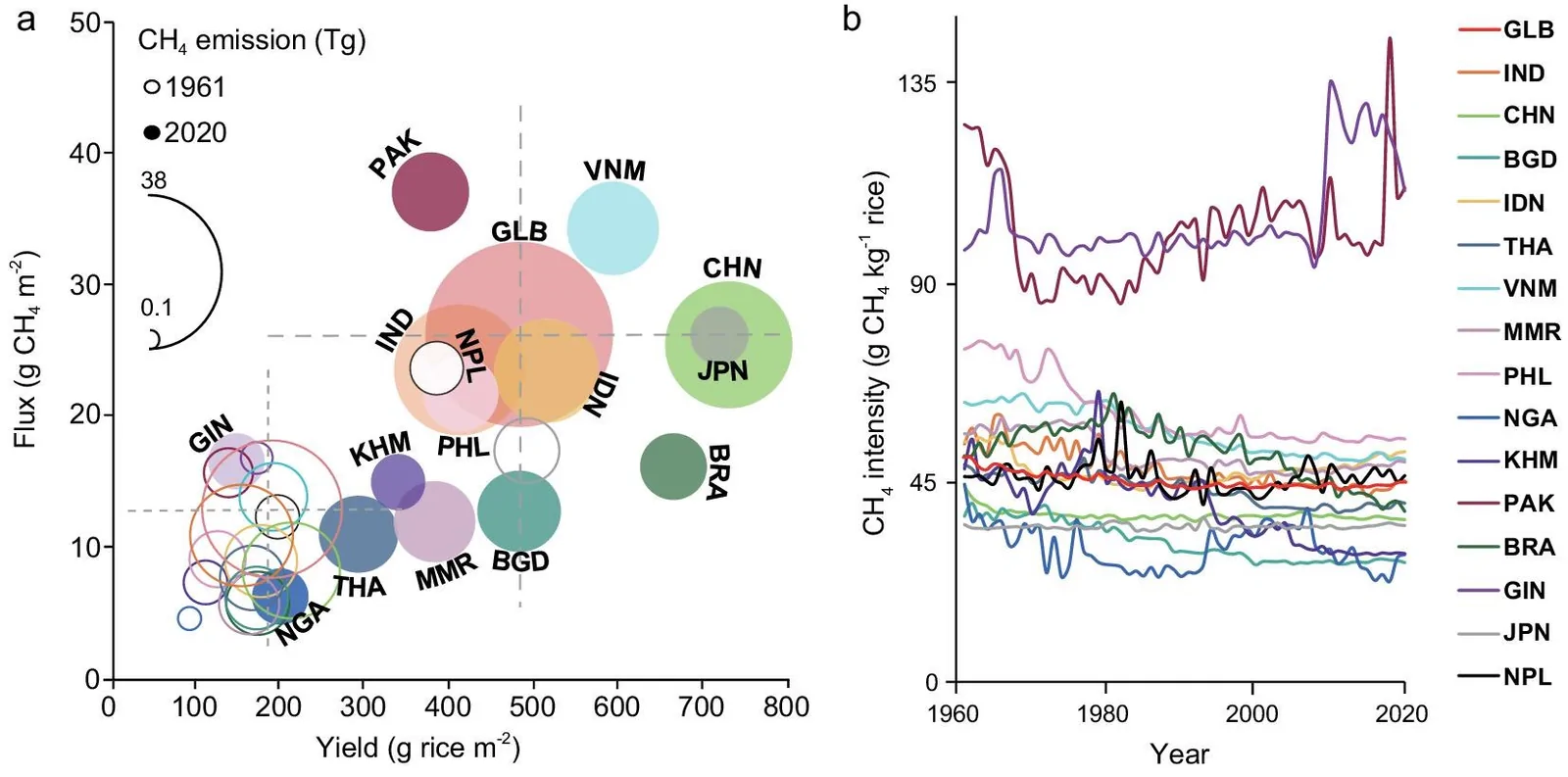
National disparities in rice yield, CH_4_ flux, and CH_4_ emission intensity across 15 major rice-producing countries. (a) Changes in rice yield and CH_4_ flux. Bubble size represents the total CH_4_ emissions from rice paddies. The dashed lines indicate the global average levels of rice yield and CH_4_ flux for 1961 and 2020. (b) Trends in CH_4_ emission intensity over 60 years and the global average [Global (GLB), India (IND), China (CHN), Bangladesh (BGD), Indonesia (IDN), Thailand (THA), Vietnam (VNM), Myanmar (MMR), Philippines (PHL), Nigeria (NGA), Cambodia (KHM), Pakistan (PAK), Brazil (BRA), Guinea (GIN), Japan (JPN), Nepal (NPL)].

Country-level patterns reveal pronounced disparities in the balance between yield and emissions (Fig. 4a). Notably, China, Brazil, Bangladesh, and Indonesia fall within the high yield, low-flux quadrant, indicating relatively favorable combinations of rice productivity and CH_4_ emissions per unit area. Bangladesh achieved the lowest emission intensity at 27 g CH_4_/kg rice (Fig. 4b). Although Japan contributes only a small share of global CH_4_ emissions, it consistently maintains the highest rice yields alongside CH_4_ fluxes close to the global average (Fig. 4a), providing a useful reference case for an efficient production-GHG system. India, a key rice producer, has an emission intensity of ∼45 g CH_4_/kg rice, comparable to the global average (Fig. 4b).

Beyond individual country performance, emission intensity shows a highly uneven global distribution of rice production. In the 2010s, 44% of global rice production was produced in countries with emission intensities below the global average. Among these low-intensity producers, China is the single largest contributor, accounting for 63% of total rice production within this group (Fig. S2). This concentration highlights that a substantial share of global rice supply is already produced with relatively low methane emission intensity, despite continued increases in total emissions worldwide.

In contrast, some countries display a combination of low yields and disproportionately high CH_4_ fluxes per unit area. Pakistan produces rice at only ∼78% of the global average yield, yet its CH_4_ flux is 1.4 times the global average (Fig. 4a), leading to a high emission intensity of 112 g CH_4_/kg rice, more than twice the global average (Fig. 4b). This places Pakistan in the low-yield, high-flux quadrant (Fig. 4a), signaling an urgent need and opportunity for improvement. Several emerging rice-producing countries in Africa show a similar pattern (Fig. 4a). In particular, Guinea produces rice at merely 30% of the global average yield, while its CH_4_ flux is 63% of the average, resulting in an exceptionally high emission intensity of 111 g CH_4_/kg rice (Fig. 4b). These findings underscore the large potential to decouple yield growth from excessive CH_4_ emissions by promoting efficient production systems.

Despite rising global CH_4_ emissions, the CH_4_ emission intensity has a significant downward trend worldwide. Spatially, 67% of grid cells, accounting for 71% of global rice harvested area, exhibit declining emission intensity (Table S3). Among the top 15 rice-producing countries, 9 of them exhibit statistically significant declining trends in emission intensity (*P* < 0.001), while only one shows a significant increasing trend. In Bangladesh, Vietnam, Brazil, Cambodia, the Philippines, India, and Myanmar, over 90% of rice harvested areas show declining trends in emission intensity, with national average slopes exceeding −2 g CH_4_/kg rice/decade, indicating improvements in production efficiency. Thailand and Nigeria also have consistent declines, though at a more moderate pace (−0.5 to −2 g CH_4_/kg rice/decade). China, Indonesia, Nepal, and Japan show relatively small changes in emission intensity. Especially in China and Japan, emission intensities have plateaued due to already high yield levels and limited room for further improvement. In contrast, Pakistan and Guinea buck the global trend, with more than half of their rice area showing increasing emission intensities (>+0.5 g CH_4_/kg rice/decade), largely driven by stagnating or even declining yields.

Together, these results demonstrate that agricultural efficiency, expressed as emission intensity, lies at the heart of aligning rice productivity and climate mitigation, and targeted, region-specific strategies are required to achieve simultaneous gains in food security and methane reduction.

## DISCUSSION

### Drivers and magnitude of the historical rice methane emissions

Over the past six decades, global CH_4_ emissions from rice paddies have more than tripled, reaching 38.8 Tg CH_4_ yr^−1^ by 2020 based on the Tier 3 mean. Our results demonstrate that this increase is primarily driven by the combined effects of rice area expansion and yield-driven intensification, with additional contributions from warming-induced biogeochemical feedback. Expansion of rice cultivation, largely in response to population growth and rising food demand [29], accounts for one-third of the total increase in emissions. At the same time, rising yields have increased the supply of methanogenic substrates through root exudation and residue inputs, amplifying CH_4_ production per unit area. These findings highlight the growing climate risk posed by agricultural intensification [30] and the need for mitigation strategies that reconcile food security with environmental sustainability.

A central implication of our analysis is that conventional EF-based inventories likely underestimate both the magnitude and acceleration of rice CH_4_ emissions under intensifying production systems. EF-based approaches continue to dominate national greenhouse gas inventories and international reporting frameworks because of their simplicity, transparency, and relatively low data requirements [1,8,15]. However, most existing inventories indicate relatively modest long-term growth rates (∼0.3–1 Tg CH_4_ per decade) [4–8], largely reflecting changes in harvested area. Even with higher-resolution activity data, our improved CH4-EF estimate remains lower than the Tier 3 estimates in recent decades. For 2020, the Tier 3 mean is 18% higher than CH4-EF and 36% higher than the FAO estimate. This structural divergence is also reflected in comparisons with the Global Methane Budget [3]. For the 2010s, our Tier 3 estimate (36.77 Tg CH_4_ yr^−1^) remains within the reported range for rice paddies (25–37 Tg CH_4_ yr^−1^), although it lies very close to the upper bound. This comparison should not be interpreted as direct validation, because rice emissions in current global methane budgets are largely constrained by EF-based bottom-up inventories. Static or weakly differentiated EFs may be insufficient to represent yield-driven increases in substrate availability and temperature-sensitive methanogenesis. Under continued intensification and warming, such structural limitations could increasingly hinder the accurate tracking of rice CH_4_ dynamics, potentially leading to systematic underestimation of future emissions and contributing to insufficient mitigation targets or a false sense of progress. Conversely, reliance on static EF approaches may also obscure mitigation opportunities in high-emission regions by underestimating their true reduction potential.

The robustness of the inferred emission trends is supported by the convergence of two structurally distinct Tier 3 approaches under a harmonized input framework. Despite their distinct structures, the process-based CH4MOD and the data-driven CH4-ML yield highly consistent temporal trajectories and spatial patterns. Rather than treating one model as a reference, their convergence serves as an internal consistency check that helps constrain model-related uncertainty in the absence of comprehensive global observations. Published global, regional, and national estimates further place our reconstruction within the broader range of existing studies, including rice-specific inventories, process-based simulations, upscaled products, EF-based inventories, and estimates informed by atmospheric observations (Fig. S7 and Table S6). These comparisons show that our estimates are not uniformly higher than previous studies; rather, their relative positions vary by region and comparison dataset. At the global scale and across Asia, our Tier 3 estimates are close to recent rice-specific or process-based estimates, including GRPI and Zhang *et al*. [31,32], while being higher than conventional inventories such as EDGAR, FAOSTAT, and CEDS [4,5,33]. Our global estimate is also lower than the GOSAT-based estimate reported by Lu *et al*. [34]. In China, Japan, and North America, our estimates are broadly comparable with available national or regional studies and are not consistently higher than previous estimates. In India, Indonesia, and parts of Southeast Asia, the spread among published estimates is larger. More broadly, differences among rice CH_4_ datasets can arise from variations, in activity data, management assumptions, regional boundaries, seasonal coverage, and model structure, with additional uncertainty introduced when estimates are extrapolated across spatial scales [35]. For Africa, although direct field observations remain sparse, the simulated rapid recent increase is consistent with the evidence based on atmospheric observations linking the expansion of African rice cultivation to rising methane emissions [22,31].

### Uncertainties in current studies and future needs

Several uncertainties and limitations nevertheless warrant consideration. The observation database used in this study covers countries accounting for 78% of global rice harvested area, 85% of global rice production, and 76% of estimated rice CH_4_ emissions, representing most major rice-producing and emitting regions. Field observations remain sparse in newly expanded rice-growing regions, such as Africa and South America [22], limiting model calibration and validation. Estimates for these regions are therefore more dependent on model extrapolation and on the reliability of historical harvested-area and yield statistics. In addition, the attribution of emission drivers should be interpreted as a scenario-difference analysis rather than strict causal attribution. Each driver was evaluated relative to the same reference simulation and is, therefore, not affected by the order in which drivers are introduced. However, interactions among temperature, yield, and organic matter inputs cannot be fully separated. These interactions may lead to underestimation of some contributions, particularly those related to manure application and indirect temperature effects on productivity [18,27,28].

Historical changes in water management, straw incorporation, and rice varieties represent additional sources of uncertainty. Globally consistent time-series datasets for water management and straw incorporation are not available for 1961–2020, and the baseline simulations therefore rely on static management assumptions. This limitation is potentially important because water-saving irrigation practices have expanded in some regions over recent decades, while restrictions on open-field straw burning and policies promoting residue recycling have likely increased straw incorporation in many rice-producing regions [36–39]. To assess the potential influence of these assumptions on the long-term emissions trend, we conducted CH4MOD sensitivity experiments using bracketing scenarios. At perturbation levels of 10%, 20%, and 30%, these scenarios considered wetter water management and lower straw inputs during the early period, as well as increased intermittent irrigation and straw inputs in recent decades, both individually and in combination (Table S8). The results showed that relative to the baseline simulation, water-management perturbations changed 2020 CH_4_ emissions by −5.1% to −15.3%, while straw-input perturbations changed emissions by +4.5% to +13.4%, while the combined scenario produced negligible differences (−0.9% to −4.1%) because the opposing effects of water management and straw incorporation largely offset one another (Fig. S8 and Table S9). These bracketing tests indicate that a substantial long-term emission increase persists, although its magnitude remains sensitive to assumptions about historical management. Although changes in rice varieties may also affect methane emissions [40,41], explicitly incorporating varietal shifts at the global scale remains challenging because of the complexity and limited availability of consistent varietal classification data [42,43]. Nevertheless, part of their influence is implicitly represented through changes in rice yield, which reflect the cumulative effects of varietal improvement and associated agronomic advances [44,45].

Looking forward, a key research priority is the development of temporally explicit datasets describing changes in rice management (e.g. water, residue, manure), cultivar adoption, and mitigation practices. Such information remains a major bottleneck for process-based and machine-learning approaches alike. Improved observational coverage, particularly in emerging rice-growing regions, together with advances in remote sensing and agricultural statistics, would enable more accurate attribution of emission drivers and provide a stronger basis for incorporating rice methane dynamics into future global methane budgets.

### Implications for sustainable rice production and methane mitigation

Our results reveal a key insight: while total rice CH_4_ emissions have increased rapidly, emission intensity has declined slightly at the global scale. This pattern reflects the counterbalancing effects of yield growth and per-area emission increases, highlighting emission intensity (CH_4_ emissions per unit of rice production) as a core indicator for aligning climate mitigation and food security, bridging SDG2 (Zero Hunger) and SDG13 (Climate Action). Approximately 44% of global rice production in the 2010s occurred in countries with below-average emission intensities, indicating that a substantial share of rice supply is already produced under relatively lower methane intensity. At the same time, continued growth in total emissions shows that efficiency gains alone are insufficient to offset the climate impact of expanding and intensifying rice production. Japan illustrates well that high yields, relatively low emission intensity, and declining total emissions can coexist under long-term changes in cultivation area and field management [46]. This example highlights the potential value of integrated improvements in productivity and management, but mitigation pathways will necessarily differ across regions because of variations in biophysical conditions, cropping systems, water availability, labor costs, and policy contexts.

Decoupling rice production from CH_4_ emissions will require region-specific strategies that distinguish between yield-driven efficiency gains and active mitigation. In high-intensity, low-yield regions, improving agronomic efficiency may reduce emission intensity while supporting food production. For low-intensity but high-production countries, further reductions in total emissions will likely require active mitigation measures rather than yield gains alone. Strategies such as alternate wetting and drying (AWD), optimized nutrient and residue management, and the adoption of high-yield, low-emission varieties have demonstrated success in reducing emissions without compromising productivity [47,48]. In some cases, transitioning to alternative crops or increasing rice imports from more efficient producers may also be warranted. To achieve equitable mitigation outcomes, international cooperation will be essential to facilitate technology transfer and capacity building in low-resource settings.

Our work lays the foundation for more transparent, spatially explicit, and temporally dynamic representations of agricultural CH_4_ emissions in global methane budgets. The dataset and methodological framework developed here can contribute to international assessments such as the Global Methane Budget of the Global Carbon Project. Ultimately, by embracing a multi-faceted and data-driven approach, it may be possible to mitigate the climate impact of rice cultivation while securing the necessary global food supply.

## MATERIALS AND METHODS

We quantified spatially explicit CH_4_ emissions from global rice paddies over 1961–2020 using three structurally distinct yet harmonized modeling approaches: a process-based model (CH4MOD) [49,50], a data-driven machine-learning framework (CH4-ML), and an improved emission-factor-based approach (CH4-EF). These approaches represent the major methodological frameworks currently used for rice CH_4_ estimation and were implemented with a harmonized set of gridded input data, enabling robust reconstruction of long-term emission trends and evaluation of methodological uncertainties.

CH4MOD and CH4-ML were used to develop a new observation-constrained global CH_4_ emission inventory at 5-arcmin spatial resolution. CH4MOD explicitly represents CH_4_ production, oxidation, and transport processes in rice paddies, driven by environmental conditions and agricultural management practices [50–52]. Model performance was previously validated against 986 site-level observations spanning diverse climatic zones and management regimes, demonstrating strong agreement with measurements (*R*^2^ = 0.76) [49]. The spatial distribution of the field observations is provided in the Supplementary Materials (Fig. S5). In parallel, CH4-ML employed a machine-learning framework trained on the same observational dataset, using key predictors including rice yield, water management regime, organic matter inputs, soil texture, phenology, and growing-season temperature. Candidate machine-learning models were compared, and Random Forest was selected for global application because it showed strong and stable performance, with *R*^2^ = 0.80 for the random test set and *R*^2^ = 0.67 (±0.16) under site-independent cross-validation. Details of model selection, validation design, and transferability evaluation are provided in the Supplementary Materials (Table S5 and Fig. S6).

To compare the two Tier 3 estimates with conventional inventory approaches, we implemented an improved Tier 2 method (CH4-EF) following the IPCC Guidelines. Unlike traditional national-scale implementations, CH4-EF was applied on a grid-by-grid basis using gridded harvested area, cultivation period, and IPCC-based management scaling factors consistent with those used in CH4MOD and CH4-ML. The baseline EFs were calculated using both the 2006 and 2019 IPCC parameterizations [53,54], allowing direct comparison with existing global inventories.

All three approaches were driven by a harmonized suite of high-resolution input datasets developed in this study, including daily air temperature (ERA5) [55], soil texture (HWSD) [56], rice harvested area and yield [57], cropping systems and phenology [58,59], organic matter inputs (straw and manure) [60], and water management practices. Particular emphasis was placed on reconstructing spatially explicit water management and organic matter application patterns, incorporating national statistics, subnational inventories, and literature-based constraints. Simulations were conducted at daily to seasonal time steps and aggregated to annual emissions for each grid cell.

Model outputs were analyzed consistently across methods to assess spatial patterns, long-term trends, and changes in emission fluxes and emission intensity (emissions per unit of rice production). Driver contributions were quantified using fixed-driver simulations in CH4MOD to estimate the apparent contributions of rice area, temperature, root exudates, straw incorporation, and farmyard manure application to historical CH_4_ emission changes. Additional analyses, including CH4MOD management sensitivity experiments and comparisons with published global, regional, and national estimates, were conducted to provide additional context for interpreting the robustness and relative magnitude of our estimates, with details provided in the Supplementary Materials.

## Supplementary Material

nwag555_Supplemental_File

## Data Availability

All datasets used to produce the figures and results presented in the main text are publicly available on Figshare at https://doi.org/10.6084/m9.figshare.29287904.
