# Supplementary material for: Global rice methane emissions tripled over six decades amid declining emission intensity
Source: Natl Sci Rev. 2026 Aug 29;13(19):nwag555. doi: 10.1093/nsr/nwag555 (PMC13637625; doi:10.1093/nsr/nwag555)
Supplement: Supplementary file 1 [file nwag555_supplemental_file.pdf]

Global rice methane emissions tripled over six decades amid declining emission intensity

Qiwen Hu[1,†], Tingting Li[2,3,†], Hanzhi Xie[1], Yao Huang[2,4], Josep G. Canadell[5], Jingxian Li[1], Pete Smith[6], Wenping Yuan[7], Wen Zhang[2], Jianwen Zou[8], Jing Liu[1], Jiawen Zhang[1], Zhangcai Qin[1,3,‡], Shilong Piao[7]

[1] School of Atmospheric Sciences, Guangdong Province Data Center of Terrestrial and Marine Ecosystems Carbon Cycle, Sun Yat-sen University, Zhuhai 519000, China

[2] Key Laboratory of Atmospheric Environment and Extreme Meteorology, Institute of Atmospheric Physics, Chinese Academy of Sciences, Beijing 100029, China

[3] Southern Marine Science and Engineering Guangdong Laboratory (Zhuhai), Zhuhai 519000, China

[4] State Key Laboratory of Vegetation and Environmental Change, Institute of Botany, Chinese Academy of Sciences, Beijing 100093, China

[5] Global Carbon Project, CSIRO Environment, Canberra, ACT 2601, Australia

[6] Institute of Biological and Environmental Sciences, University of Aberdeen, Aberdeen AB24 3UU, UK

[7] Institute of Carbon Neutrality, College of Urban and Environmental Sciences, Peking University, Beijing 100871, China

[8] College of Resources and Environmental Sciences, Nanjing Agricultural University, Nanjing 210095, China

[†] These authors contributed equally to this work.

[‡] Corresponding author: Zhangcai Qin, Email: qinzhangcai@mail.sysu.edu.cn

This PDF file includes:

Supplementary Materials and Methods

Supplementary Data S1-S5

Supplementary Figures S1-S8

Supplementary Table S1-S9

Supplementary References

Supplementary Materials and Methods

This study assessed spatially explicit CH_4 emissions from global rice paddies between 1961 and 2020 using three structurally distinct but harmonized approaches: (1) a process-based model (CH4MOD) [1,2], (2) a machine learning algorithm (CH4-ML), and (3) an improved emission factor-based approach (CH4-EF). CH4MOD and CH4-ML were used to develop a new, observation-constrained global CH_4 emission inventory for 1961–2020. This inventory incorporates spatial heterogeneity and temporal dynamics in environmental and management drivers, enabling robust assessment of long-term emission trends and growth rates. The CH4-EF method refines traditional Tier 1 approaches by replacing national-scale scaling coefficients with grid-specific estimates, enhancing spatial representativeness. It was used to generate a benchmark inventory for comparison with conventional EF-based estimates, providing an independent line of evidence to evaluate the reliability of our improved inventory. All three methods were driven by harmonized inputs derived from a suite of high-resolution, spatially explicit input datasets developed in this study, covering climate and soil conditions, rice cultivation information, and anthropogenic management practices (Table S4), ensuring consistency in spatial coverage and model comparability. Simulations were conducted at 5 arcminute spatial resolution and aggregated at annual scales.

Data

This study utilized a comprehensive, long-term, and high-resolution dataset to simulate global CH_4 emissions from rice paddies, incorporating key biophysical and management variables. The model inputs included atmospheric temperature, soil properties, rice harvested area, crop yield, crop phenology, crop rotation systems, exogenous organic matter addition, and water management practices. The datasets used for the three modeling approaches are summarized in Table S4.

Daily air temperature data were sourced from the ERA5 reanalysis dataset (0.25° resolution, 1961–2020) [3] and resampled to a 5-arcminute resolution using nearest-neighbor interpolation. Soil sand content were obtained from the HWSD V2.0 database (30-second resolution) [4] and aggregated to 5-arcminute resolution by averaging values within each grid cell.

Rice harvested area data were compiled from a previously developed global dataset, GloRice-hvst-Intensive dataset from Xie et al. [5], which provides 5-arcminute annual gridded estimates of rice harvested area from 1961 to 2020. The methodology for crop yield followed a similar logic, integrating geographic distribution data [6] with FAO national statistics [7]. Where area data existed without corresponding yield data, country-level averages were applied.

Cropping rotations were obtained from the global cropping system dataset [8], provided at a 0.5° resolution. Rare triple-cropping systems were reclassified as double systems, as they account for only approximately 3.5% of the total harvested rice area globally. Crop phenology was primarily based on provincial-level data from RiceAtlas [9]. To resolve discrepancies between phenological data and cropping system classifications, crop rotation information was prioritized. Both datasets were downscaled to 5-arcminute resolution using the nearest-neighbor method to align with the spatial resolution of other inputs. A hierarchical matching scheme was implemented, prioritizing data from adjacent provinces with similar latitudes, terrain, or geographic proximity. The resulting dataset represents global phenological distributions for single and double rice cropping systems (Figure S3).

Exogenous organic matter addition included manure and straw inputs. Manure inputs were estimated by combining the 5-arcminute manure nitrogen dataset from Zhang et al. [10] with the cropland fraction from Ramankutty et al. [11]. We assumed a carbon-to-nitrogen ratio of 20 [12] and a carbon content of 40% in manure [13]. Straw input included residues from previous crops (roots and stubble) and straw incorporation. Single rice cropping systems were categorized into five major rotation types based on

Waha et al. [8]: rice–rapeseed, rice–wheat, rice–maize, rice–soybean, and rice–other crops. In multi-cropping systems, straw was assumed to originate solely from rice. Crop-specific straw amounts were calculated using economic coefficients and yield data, while straw incorporation was determined based on incorporation rates. Root biomass was estimated using the root-to-shoot ratio, and stubble residue was assumed to account for 10% of the straw. The economic coefficients and root-to-shoot ratios were primarily based on national-scale data provided by Han et al. [14]. For China, straw incorporation rates were obtained from The Third National Communication on Climate Change of the People's Republic of China [15], which includes provincial-level straw incorporation rates. For other major rice-growing countries in the Asia-Pacific region, data were sourced from relevant references published by the United Nations Center for Sustainable Agricultural Mechanization (ESCAP-CSAM) [16]. For the remaining countries, straw incorporation rates were assumed to be 30% for developing countries and 70% for developed countries [14]. These data were downscaled to 5-arcminute resolution by assigning each grid cell within a given country or province the same value as that of the corresponding administrative unit.

Water management data were developed by integrating multiple data sources, preprocessing steps, and refining spatial resolution to produce a globally provincial-scale distribution dataset. The primary data inputs included national water management data [17], providing proportions of irrigated, upland, and rainfed rice fields for each country, and ALGAS data [18], which detailed the proportions of continuous flooding (CF), single drainage (SD), and multiple drainage (MD) practices in major rice-growing regions. For China, provincial-level data from the 2010 National Methane Inventory [15] were used to achieve finer resolution. Additionally, literature and statistical data on traditional irrigation practices were incorporated to refine classifications for specific regions. In the preprocessing stage, national data from IPCC [17] and ALGAS [18] were extracted, with provincial-scale data prioritized where available. Literature-based classifications were applied in regions with detailed descriptions of traditional irrigation

practices. Monthly water depth during the rice-growing season was estimated based on soil saturation capacity and actual water content, providing a basis for distinguishing CF, SD, and MD practices. To allocate these practices spatially, national-level CF, SD, and MD shares were distributed to provinces in accordance with observed irrigation patterns. CF and SD were matched to CH4MOD regimes F and FDF, while MD was further subdivided based on water depth variability and region-specific practices. The resulting dataset captures spatial and temporal heterogeneity in water management at an unprecedented resolution, enhancing the accuracy of CH_4 emission modeling (Figure S4).

Modeling

The IPCC guidelines recommend a tiered approach for greenhouse gas emissions estimation [19]: (1) Tier 1 uses default emission factors for all parameters; (2) Tier 2 incorporates country-specific data to develop more accurate emission factors; (3) Tier 3 involves advanced models or monitoring networks for high-resolution estimates. To comprehensively assess CH_4 emissions from rice paddies, we applied two Tier 3 approaches, a process-based model and a data-driven machine learning method, and used an enhanced Tier 2 emission factor approach as a benchmark.

For the process-based model, CH4MOD was chosen for its ability to simulate CH_4 production, oxidation, and emissions in rice paddies under various environmental conditions and agricultural management practices [2, 20, 21]. The CH4MOD model was globally validated against 986 observations in our previous study, covering diverse environmental and management conditions, achieving a robust correlation coefficient (R^2) of 0.76 and a high model efficiency of 0.78, indicating excellent overall performance across global rice systems [1]. The results confirmed the reliability and applicability of CH4MOD across different scales and regions.

The model simulates CH_4 production as a function of methanogenic substrate availability (from root exudates and added organic matter) and environmental drivers

(soil temperature, texture, and redox potential). CH_4 transport via rice aerenchyma and ebullition, while oxidation occurs mainly during plant-mediated transport, increasing over the crop growth period. Model inputs include daily air temperature, soil sand content, rice grain yield, type and amount of organic matter amendment (manure, straw, or stubble residues), and water management regime (six irrigated and two rainfed types). More details on the model are described in previous studies [1, 2, 22, 23]. The model outputs gridded daily CH_4 fluxes, which are summed over the entire growing season and multiplied by the corresponding harvested area to derive annual emissions.

For machine learning, we chose three tree-based ensemble models (Random Forest, Extra Trees, and XGBoost), as they are well-suited to capture complex, non-linear interactions among variables [24, 25]. Model inputs mirrored those used in CH4MOD and included: rice yield, water regimes, organic matter amendments, soil sand content, and length of the cultivation period (Phenology). The only exception was temperature: while CH4MOD used daily values, the machine learning models used accumulated growing-season temperature. Water regimes were treated as a categorical variable, while all other features were numerical.

The models were trained and validated using the same dataset of 986 observations used to evaluate CH4MOD (Figure S5)[1]. The dataset was randomly divided into a training set (80%) and a testing set (20%), with hyperparameters optimized through Grid Search combined with 5-fold Cross-Validation [26, 27]. To evaluate model robustness and transferability beyond a single random split, model performance was further assessed using 100 repeated random 80/20 train–test splits and five-fold site-independent cross-validation, in which observations from the same geographical site were assigned to the same fold [28, 29]. Random Forest was selected for global application because it showed the best performance under site-independent cross-validation among the candidate models, while maintaining strong performance under random validation and avoiding the negative predictions generated by XGBoost (Table S5; Figure S6). Based on a global 5-arcminute input dataset, Random Forest estimated

seasonal CH_4 fluxes for each grid cell, which were then multiplied by the harvested area to compute total emissions, collectively denoted as CH4-ML.

For EF-based approach, we followed the Intergovernmental Panel on Climate Change (IPCC) Guidelines, which applied a global default baseline emission factor (EF_c, for continuously flooded fields without organic amendments) with scaling factors (SF_s) that adjust for specific conditions such as water regimes during cultivation (SF_w), pre-cultivation water regimes (SF_p), and type and amount of organic amendments (SF_o) [19]. In the 2019 refinement [30], IPCC introduced an updated categorization for pre-season water regimes and revised the EF_c and SF values. Specifically, EF_c values were differentiated by continent instead of a single global default, and SF_w values were modified. SF_p and SF_o retained the same classification structure and values [30]. Most existing global CH_4 emission inventories apply region-specific emission factors or simplified categorical assignments of water and straw management practices (Table S1).

We applied a Tier 2 EF approach in this study, using a global 5-arcminute grid dataset to calculate CH_4 emissions. All input data, including phenology, water regimes during the growing season, the amount of returned straw, and farmyard manure application, were consistent with the CH4MOD input data to ensure consistency between methods. For organic amendments, only straw (incorporated shortly (<30 days) before cultivation) and farmyard manure were considered. For each cell, the emission factor, which represents the daily CH_4 flux, was calculated using both the 2006 and 2019 IPCC guidelines [19, 30], incorporating their respective EF_c and SF values. Annual CH_4 emissions were estimated by multiplying daily emission factors by the cultivation period of rice and the annual harvested area, denoted as CH4-EF.

Model evaluation and external comparison

We evaluated model performance and compared our estimates with additional lines of evidence, including field observations, stricter machine-learning validation schemes, and published country-scale or larger regional estimates.

CH4MOD has been previously evaluated against the global field-observation database used in this study, which includes 986 site-level seasonal CH_4 flux observations across diverse climatic, soil, and management conditions [1]. The model achieved an overall R^2 of 0.76 and a model efficiency of 0.78, supporting its applicability across major rice-growing systems [1]. For CH4-ML, candidate machine-learning models were evaluated using random train–test splitting, repeated random validation, and site-independent cross-validation. In the site-independent validation, observations from the same geographical site were assigned to the same fold to reduce the potential overestimation of model skill caused by site-level or spatial autocorrelation. The performance of candidate machine-learning models and the transferability assessment of the selected CH4-ML (Random Forest model) are summarized in Table S5.

We also assessed the spatial representativeness of the field-observation database. Although observations are unevenly distributed, countries represented by field observations account for 78% of global rice harvested area, 85% of global rice production, and 76% of estimated rice CH_4 emissions. This indicates that the observational database covers most major rice-producing and emitting regions, although uncertainties remain higher in underrepresented regions, particularly parts of Africa and South America (Figure S5).

Finally, we compared our estimates with published country-scale and larger regional estimates, including rice-specific inventories, regional upscaling products, process-based simulations, emission-factor inventories, and atmospheric-inversion-based studies. These comparisons are intended to contextualize our estimates rather than to serve as formal validation, because the available studies differ substantially in

spatial and temporal coverage, input data, management assumptions, and model structure. As these differences often occur simultaneously and are not always documented in sufficient detail, we did not attempt to attribute inter-study discrepancies to individual factors. The published estimates used for comparison and the regional comparison results are summarized in Table S6 and Figure S7.

Analysis

Emission trend and intensity analyses

We conducted a comprehensive assessment of the spatial and temporal patterns of rice paddy CH_4 emissions derived from the three modeling approaches under a harmonized input framework. All outputs were harmonized to a global 5-arcminute spatial resolution and annual temporal resolution; variable-specific regridding methods are detailed in the Data section. For decadal trends, emissions in the 1960s were represented by the mean values from 1961 to 1970, ensuring temporal consistency over the full study period (1961–2020).

To place our model-based estimates in the broader context of existing assessments, we compared our results against several widely used global inventories of CH_4 emissions from rice cultivation, including datasets from the Global Methane Budget [31], FAO [7], EDGAR [32], US EPA [33], GAINS [34], and Wang et al. [35]. These inventories adopt Tier 1 emission factor (EF)-based approaches following the IPCC guidelines, typically applying default or regionally differentiated EFs to aggregated national statistics on rice cultivation. We summarized the differences in methodological frameworks, spatial granularity, input datasets, and emission drivers (Table S4). This provides a basis for interpreting divergence in estimates, especially in recent decades, when rice production systems have undergone significant changes.

Our analysis focused on global as well as the top 15 rice-producing countries, which together account for nearly 90% of the global rice paddy area. This approach allowed for a comprehensive understanding of global CH_4 emission trends and hotspots.

We paid particular attention to three key indicators of change: total emissions, emission change rate, and emission change multiple, in order to further analyze the emission data. These indicators enabled us to quantify the magnitude of emissions changes and capture the temporal trends in CH_4 emissions across different regions.

Additionally, we analyzed CH_4 emissions from multiple perspectives, including total emissions, emissions flux (i.e., emissions per unit area of rice paddy), and emissions intensity (i.e., emissions per unit rice production). For regional-scale analysis at the national and global levels, emission flux and rice yield were calculated as area-weighted means, whereas emission intensity was calculated as the ratio of total CH_4 emissions to total rice production.

Driver attribution and management sensitivity tests

We used CH4MOD to evaluate the apparent contributions of major drivers to the long-term changes in rice CH_4 emissions. Five drivers were considered: rice harvested area, temperature, straw incorporation, root exudates, and farmyard manure application. For each driver, we conducted a fixed-driver simulation in which the focal driver was held at its 1961 level, while all other drivers varied annually over 1961–2020 as in the reference simulation. The difference between the baseline simulation and the corresponding fixed-driver simulation was used to estimate the apparent contribution of that driver. The residual term was calculated as the difference between the total simulated emission change and the sum of the apparent contributions from the five individual drivers, and was reported separately in Table S7.

Because each driver was evaluated separately against the same reference simulation rather than introduced sequentially, the results are not sensitive to the order of driver introduction. The apparent effects were expressed as percentages to compare the relative importance of different drivers. However, CH_4 emissions respond nonlinearly to environmental and management variables and interactions among drivers

cannot be fully separated. The results were therefore used to identify dominant drivers of long-term emission changes rather than to provide a strict causal decomposition.

We further conducted management sensitivity experiments to test whether the long-term emission trend was robust to plausible deviations from the baseline assumptions for water management and straw incorporation. Because globally consistent historical datasets for rice water management and straw incorporation are unavailable for 1961–2020, these experiments were designed as bracketing sensitivity tests rather than reconstructions of actual historical management trajectories. Four scenario types were considered: the baseline CH4MOD simulation (BASE), water-management sensitivity (WAT), straw-incorporation sensitivity (STR), and combined management sensitivity (COM), with perturbation levels of 10%, 20%, and 30% applied to the three sensitivity scenarios (Table S8). In WAT, the corresponding proportion of rice area was shifted to fully flooded water management (F) during 1961–1980 and to intermittent irrigation (M) during 2001–2020. In STR, straw input was reduced during 1961–1980 and increased during 2001–2020 by the corresponding perturbation level. In COM, the water-management and straw-input perturbations were applied simultaneously. The intermediate period (1981–2000) was kept unchanged in all scenarios. The resulting global emission trajectories and summary statistics are shown in Figure S8 and Table S9.

Statistical metrics

We used a consistent set of statistical metrics to quantify emission changes, emission intensity, model performance, and inter-method differences.

For country-level and global emission changes, the absolute change in CH_4 emissions from 1961 to 2020 was calculated as:

$$\Delta Emi = Emi_{2020}^{Coun} - Emi_{1961}^{Coun}$$

where Emi_{1961}^{Coun} and Emi_{2020}^{Coun} are annual CH_4 emissions in 1961 and 2020, respectively.

The proportional contribution of each country to the global emission increase was calculated as C_i. The emission growth factor was calculated as G_i. For the Tier 3 estimate, annual CH_4 emissions were first calculated as the arithmetic mean of CH4MOD and CH4-ML for each year. The Tier 3 growth factor was then calculated from these mean emissions in 2020 and 1961, rather than by averaging the growth factors derived separately from CH4MOD and CH4-ML.

$$C_i = \frac{Emi_{2020}^{Coun} - Emi_{1961}^{Coun}}{Emi_{2020}^{Global} - Emi_{1961}^{Global}} \times 100\%$$

$$G_i = \frac{Emi_{2020}^{Coun}}{Emi_{1961}^{Coun}}$$

Linear trends were estimated using ordinary least-squares regression over 1961–2020. The statistical significance of each trend was evaluated using the p value of the regression slope.

Model performance was evaluated using the coefficient of determination (R^2), root mean square error (RMSE), and mean absolute error (MAE):

$$R^2 = \left(\frac{\sum_{i=1}^{N}(y_i - \bar{y})(f_i - \bar{f})}{\sqrt{\sum_{i=1}^{N}(y_i - \bar{y})^2} \sqrt{\sum_{i=1}^{N}(f_i - \bar{f})^2}} \right)^2$$

$$RMSE = \sqrt{\frac{1}{N}\sum_{i=1}^{N}(f_i - y_i)^2}$$

$$MAE = \frac{1}{N}\sum_{i=1}^{N}|f_i - y_i|$$

where y_i, $\bar{y}$,f_i and $\bar{f}$ are observed CH_4, mean observed CH_4, simulated CH_4, and mean simulated CH_4, respectively.

Supplementary Data S1–S5 are provided via Figshare:

https://doi.org/10.6084/m9.figshare.29287904.

Data S1. Global CH_4 emissions from 1961 to 2020 estimated by CH4MOD, CH4-ML, CH4-EF, and other studies. Related to Figure 1a. (.csv)

Data S2. Grid-level Pearson correlation coefficients (r) between CH4MOD and CH4-ML CH4 emission time series over 1961-2020. Spatial distribution of differences between the Tier 3 mean (average of CH4MOD and CH4-ML) and CH4-EF estimates during 2010s. Related to Figure 1b-c. (.tif)

Data S3. Spatial distribution of CH_4 emissions in 2010s and the changes relative to the 1960s. Related to Figure 2a-b. (.tif)

Data S4. Annual CH_4 emissions in global total and 15 major rice-producing countries from 1961 to 2020. Related to Figure 2c. (.csv)

Data S5. Change of rice harvested area and CH_4 flux in the 2010s relative to the 1960s. Related to Figure 3a-b. (.tif)

Supplementary Figures

Figure S1 Trends in rice harvested area and yield from 1961 to 2020 across the world and 15 major rice-producing countries. (a) Rice harvested area. (b) Rice yield. (c) Country-level time series of rice harvested area (left axis) and yield (right axis). (Global (GLB), India (IND), China (CHN), Bangladesh (BGD), Indonesia (IDN), Thailand (THA), Vietnam (VNM), Myanmar (MMR), Philippines (PHL), Nigeria (NGA), Cambodia (KHM), Pakistan (PAK), Brazil (BRA), Guinea (GIN), Japan (JPN), Nepal (NPL)).

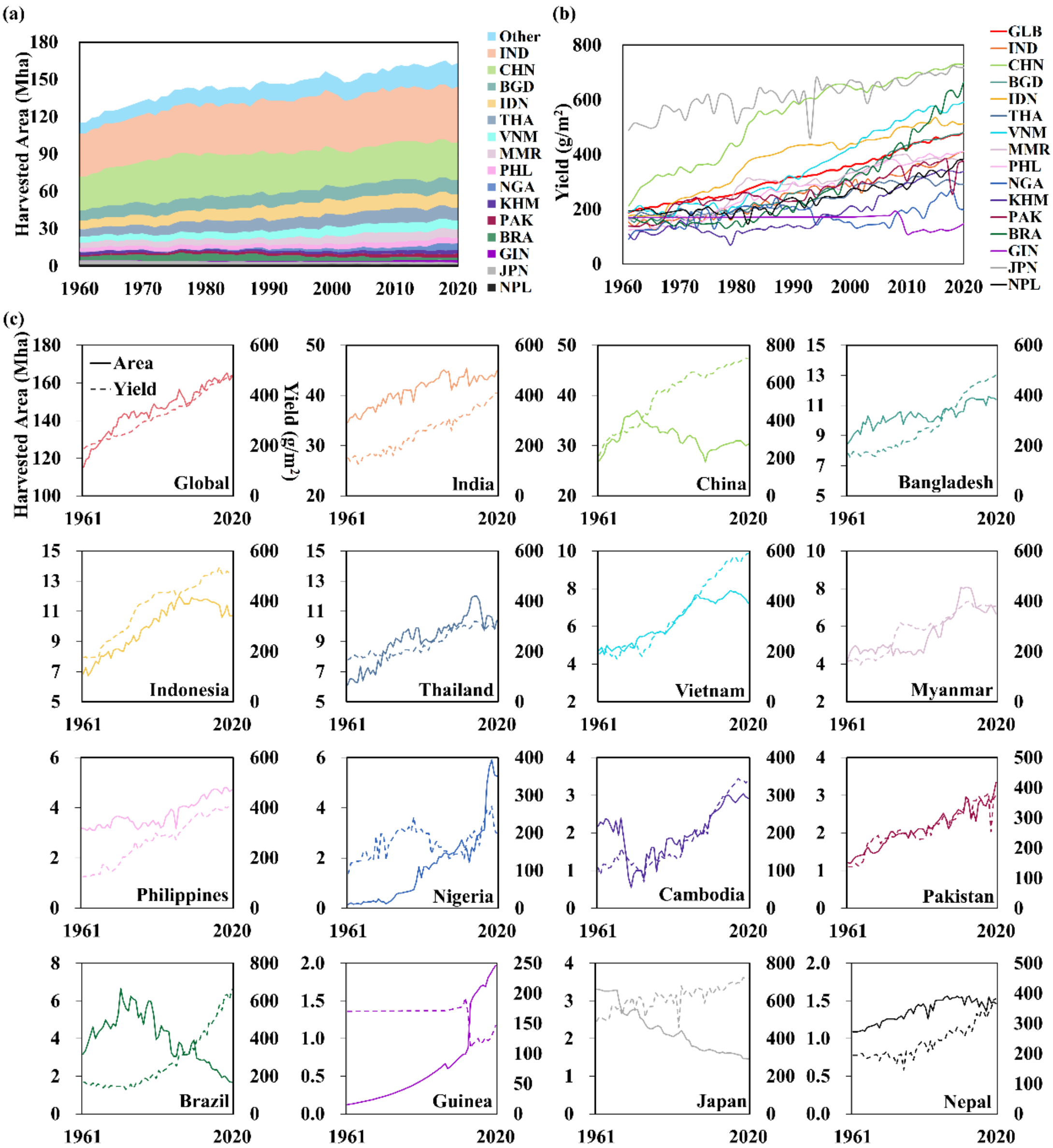

Figure S2. Cumulative distribution of global rice production ranked by national CH4 emission intensity in the 2010s.

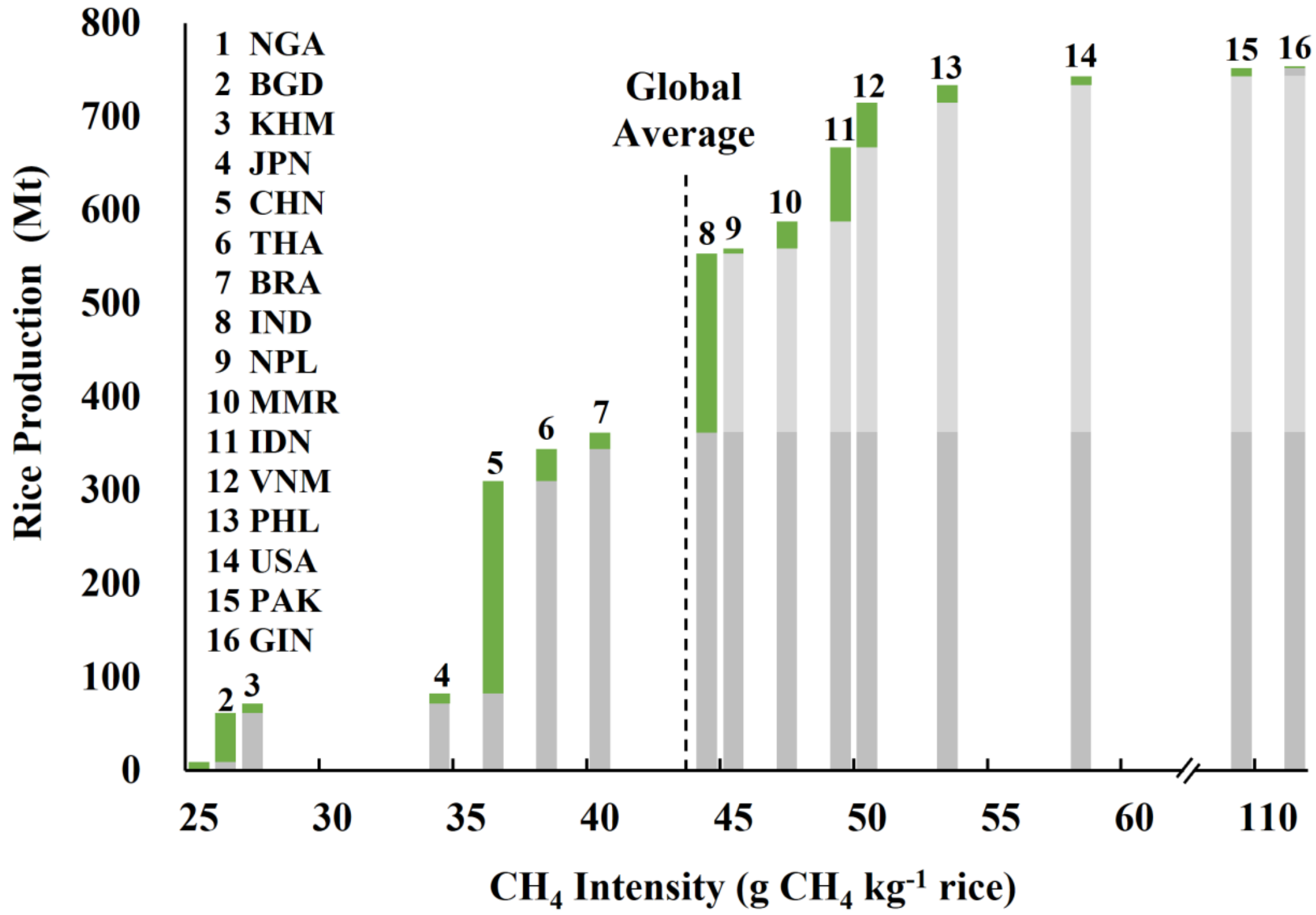

Countries are ordered from low to high by average emission intensity (g CH_4 kg^{-1} rice). The green segment denotes each country's production, while grey segments indicate cumulative production of preceding countries. The dashed line marks the global mean intensity; dark and light grey shading represent production below and above the global average, respectively.

Figure S3. Global phenological distributions for single and double rice cropping systems (Rare triple-cropping systems were reclassified as double systems.)

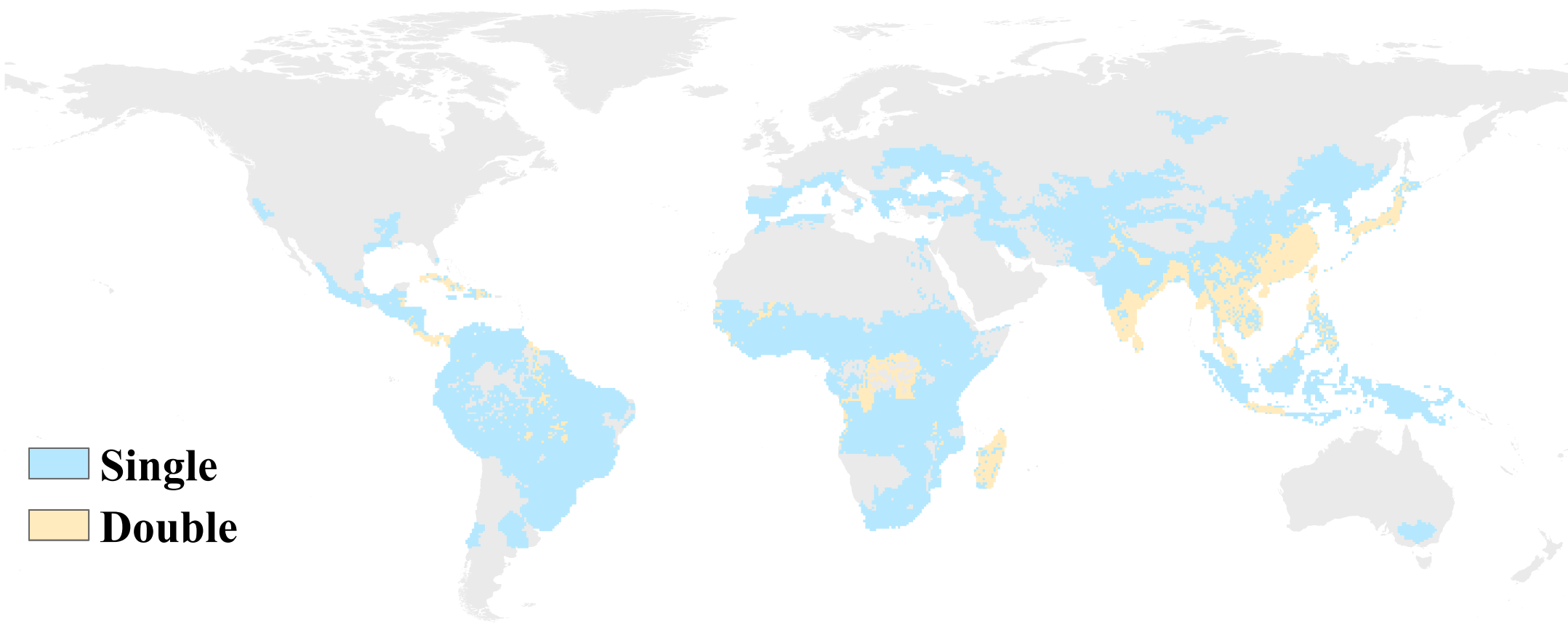

Figure S4. Global spatial distribution of water regimes. The categories F, FDF, FDFM, FDM, FM, M, RFW, and RFD represent different water management strategies: F (Flooded), FDF (Flooded-Drained-Flooded), FDFM (Flooded-Drained-Flooded-Moist), FDM (Flooded-Drained-Moist), FM (Flooded-Moist), M (Moist with Intermittent Irrigation), RFW (Rainfed-Wet Season), and RFD (Rainfed-Dry Season).

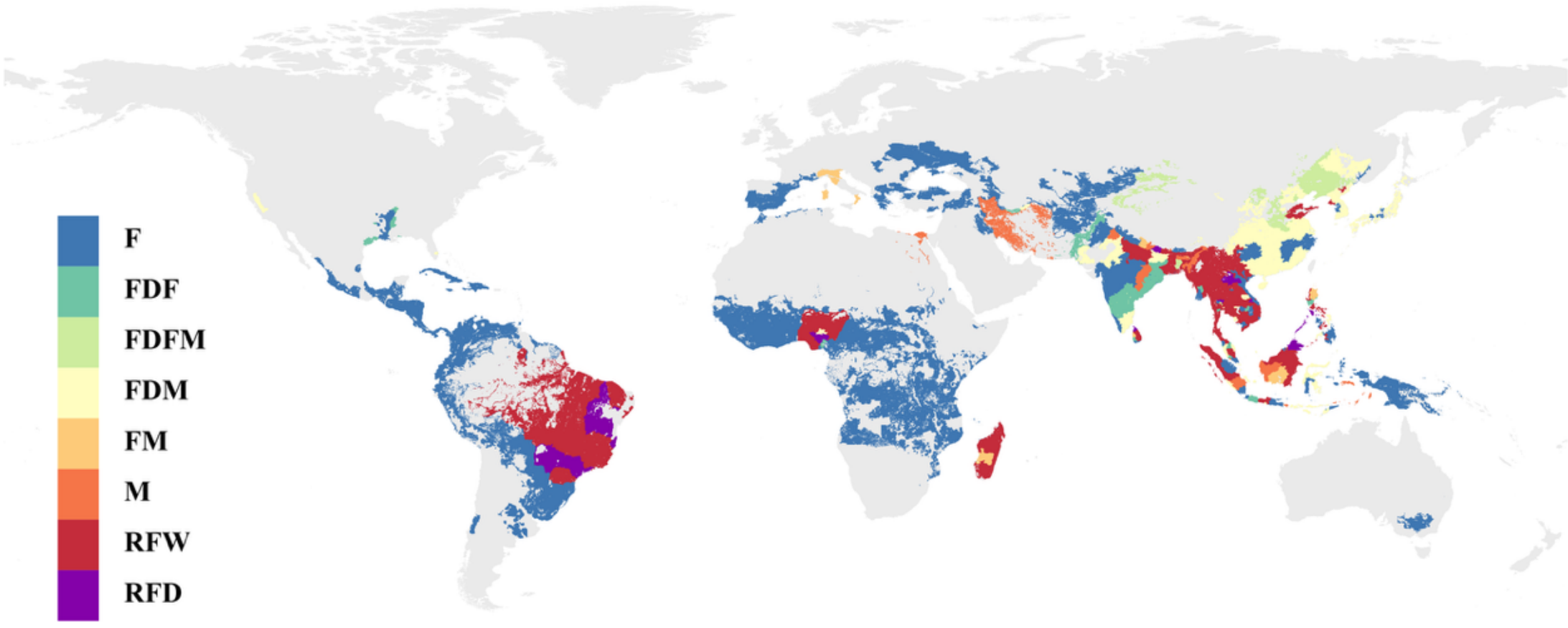

Figure S5. Distribution of rice paddy sampling sites used in the modeling. The size of the red dots reflects the number of experiments conducted at that site. The base map shows the average fractional proportion of the harvest area to the grid area from 1997 to 2003.

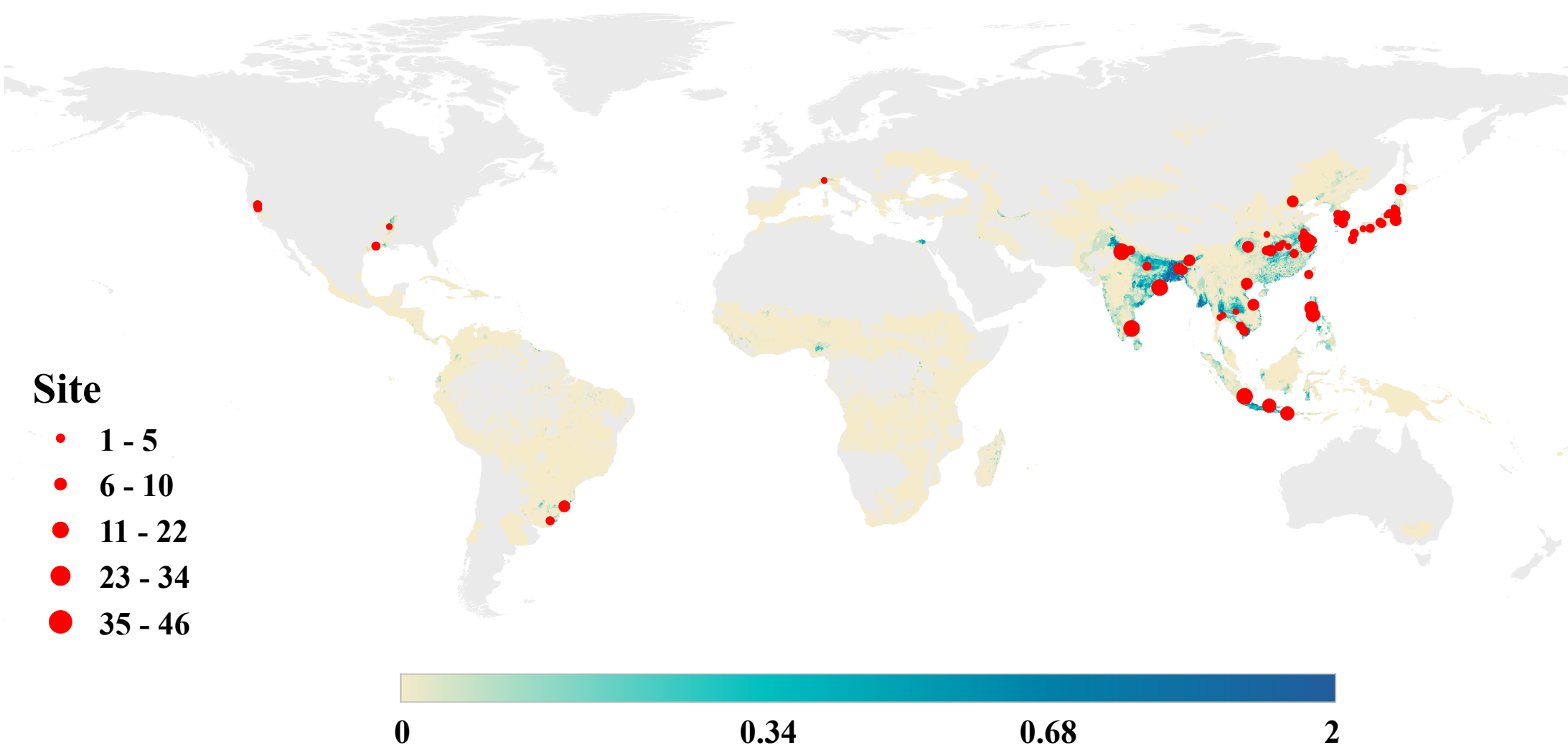

Figure S6. Performance and predictor contributions of the CH4-ML (Random Forest). (a) Comparison between observed and simulated CH_4 fluxes for the random test set from a single 80/20 train–test split (n=191, corresponding to the held-out 20% test observations). (b) Comparison between observed and simulated CH_4 fluxes under five-fold site-independent cross-validation (n=954). Observations from the same geographical site were assigned to the same fold, and predictions from all held-out folds were pooled so that each observation was predicted once when its site was held out. (c) Predictor contributions ranked by mean absolute SHAP values, which summarize the contribution of each predictor to CH4-ML predictions.

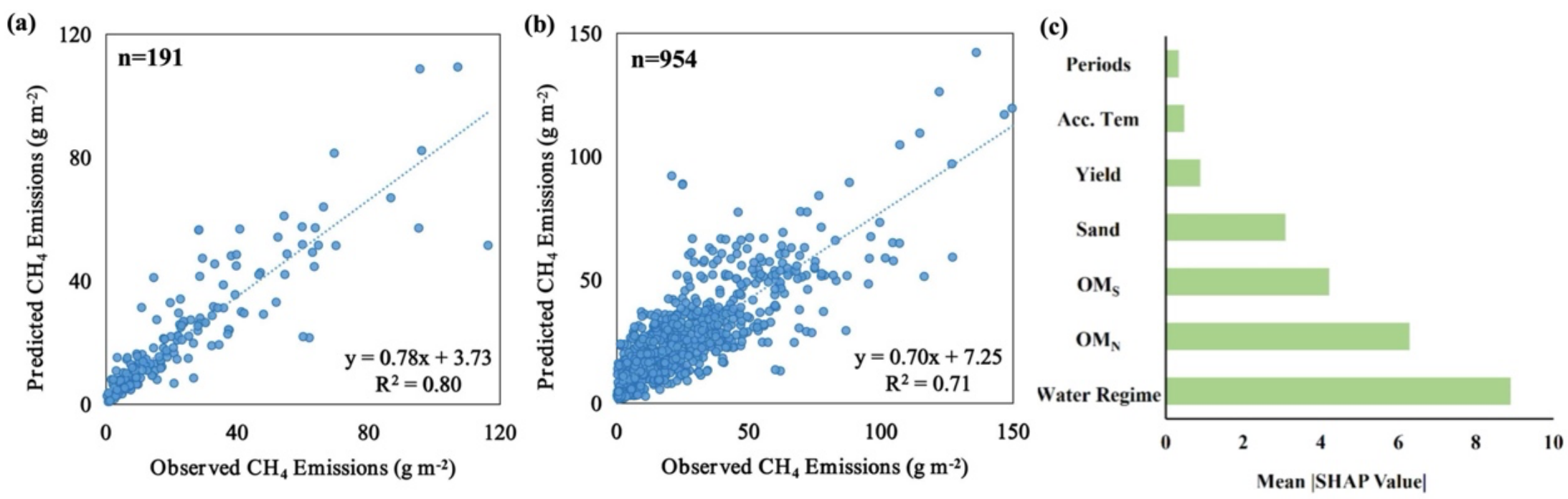

Figure S7. Comparison of rice CH_4 emission estimates from this study with published global, regional, and national estimates. Details of the compared studies and datasets are summarized in Table S6.

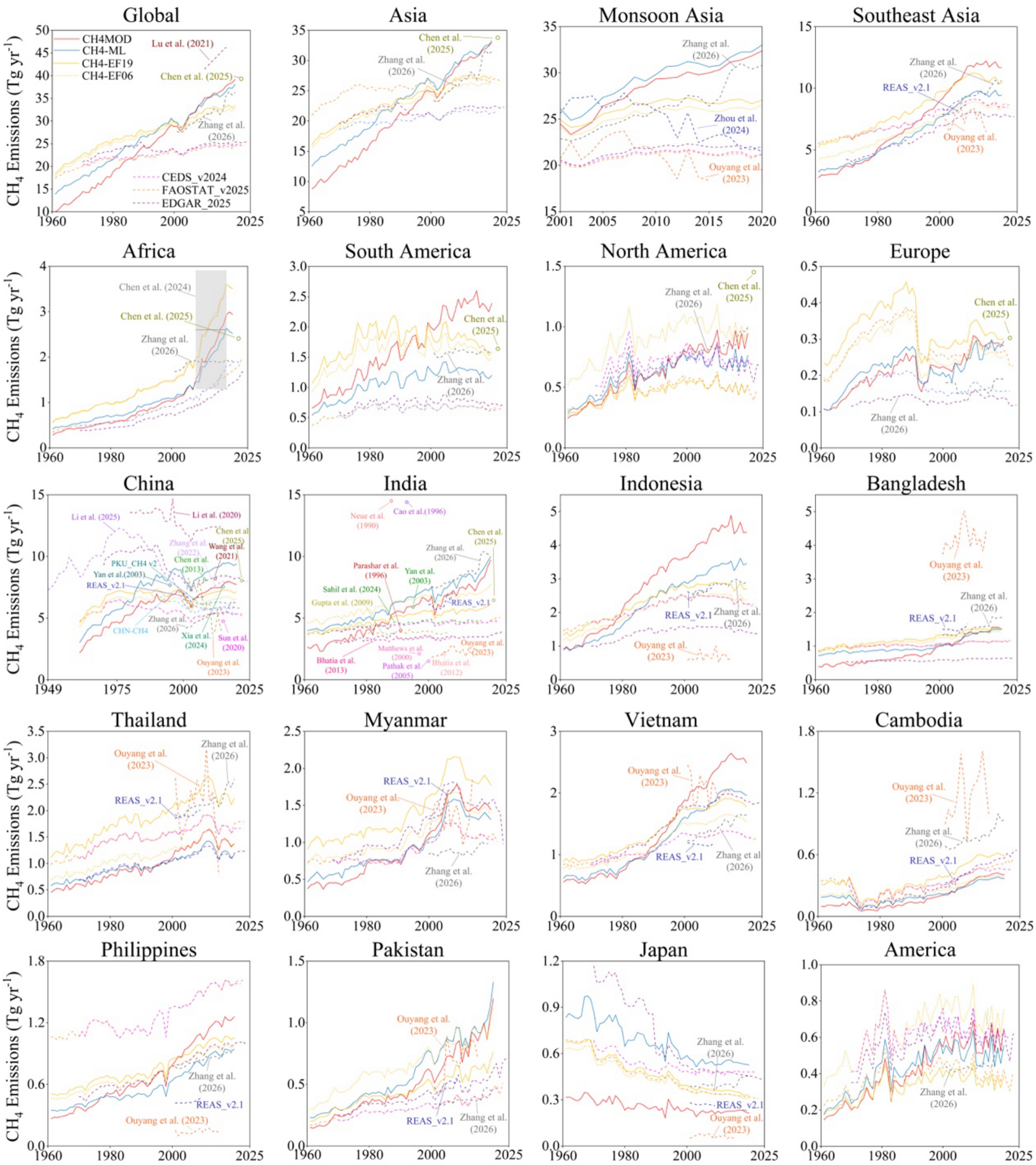

Figure S8. Global rice CH4 emissions under CH4MOD management sensitivity scenarios from 1961 to 2020. BASE represents the original CH4MOD simulation; WAT, STR, and COM represent the water-management, straw-incorporation, and combined management sensitivity scenarios, respectively, each tested at perturbation levels of 10%, 20%, and 30%. Scenario definitions are provided in Table S8.

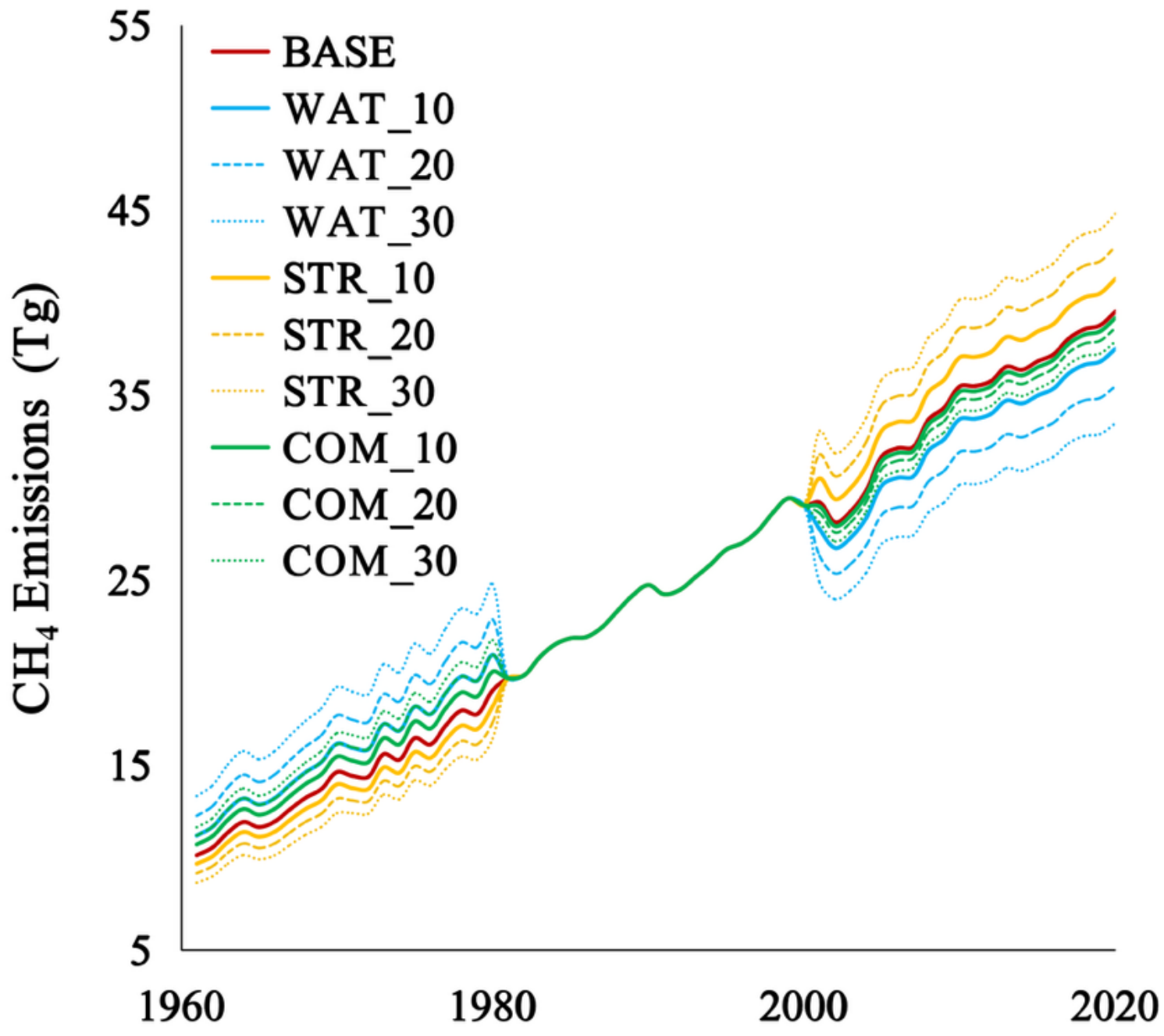

Supplementary Tables

Table S1 Summary of parameters for major emission-factor-based inventories of rice CH_4 emissions.

Inventory	EF_c	SF_p	SF_w	SF_o	A	T
FAO[7] 1961-2020	10 country-specific Others default[17,19]	Default[17,19]	Default[17,19]	Global default[17,19]	Annual, Country[7]	Default[17,19]
EDGAR v8.1[32] 1970-2020	Partly country-specific (GAINS model) Others default[19] China specific[36]	Default[19]	Default[17,37] (3 types)[17]	Global default[19]	Annual, 0.1°[7,38]	Default[19]
US EPA[33] 1990-2020 (every 5 years)	Regional-specific[19]	Default[19]	Partly country-specific[39] (4 types)[19]	Default[19]	Annual Country[7]	Partly country-specific[39]
GAINS[34] 1990-2020 (every 5 years)	Partly country-specific (GAINS model) Others default[19]	Default[19]	Partly country-specific[37] (4 types)[17]	Default[19]	Annual Country[7]	185 days[34]
Wang et al.[35] 1990-2020	Main country-specific[36] Others default[30]	Default[30]	Partly country-specific[38-40] (4 types)[30]	Partly country-specific[41]	Annual Country[7]	Country-specific[42]
CH4-EF[43] 1961-2020	Regional-specific[19.30]	Default[19.30]	Province-specific[17] (4 types)[19.30]	Annual Grid-specific[17] (2 types)[19.30]	Annual, 5 arcmin[5]	Province-specific[43]

Note:

All inventories in the table above use the emission factor (EF) method to estimate methane emissions from rice paddies. The general calculation formula is:

$$EM_{CH_4} = EF \times A \times T \quad (1)$$

$$EF = EF_c \times SF_p \times SF_w \times SF_o \quad (2)$$

where EM_{CH_4} is the annual CH_4 emissions from rice paddies (kg CH_4/yr), EF is the daily emission factor (kg CH_4/ha/day), A is the activity data, representing the annual harvested rice area (ha), T is the cultivation period of rice (days). EF_c is the baseline emission factor, representing daily CH_4 emissions per hectare under continuously flooded conditions without organic amendments. SF_p is the scaling factor for water management before the cropping season. SF_w is the scaling factor for water management during the cropping season. SF_o is the scaling factor for the type and amount of organic amendments applied.

Table S2. CH_4 emissions from rice paddies from 1961 to 2020: Global changes and those in the top 15 rice-producing countries, reported as the Tier 3 mean with the CH4MOD–CH4-ML range in parentheses. Calculation formulas are provided in the Statistical metrics section of the Supplementary Materials and Methods.

Country	Emissions in 1961 (Tg)	Emissions in 2020 (Tg)	Absolute Changes (Tg)	Proportional contribution (%)	60-yr growth (times)
Global	12.02 (10.13–13.91)	38.80 (38.07–39.53)	26.78 (24.16–29.40)	—	3.23 (2.74–3.90)
India	3.35 (2.65–4.05)	9.92 (9.71–10.13)	6.56 (6.06–7.06)	24.51 (24.03–25.11)	2.96 (2.49–3.67)
China	2.61 (2.19–3.03)	8.56 (7.73–9.39)	5.95 (5.53–6.37)	22.22 (18.84–26.34)	3.28 (3.10–3.52)
Bangladesh	0.54 (0.39–0.69)	1.47 (1.46–1.48)	0.93 (0.76–1.10)	3.48 (3.16–3.74)	2.73 (2.11–3.85)
Indonesia	0.90 (0.89–0.91)	3.93 (3.46–4.40)	3.03 (2.58–3.48)	11.31 (10.67–11.85)	4.35 (3.90–4.80)
Thailand	0.51 (0.45–0.57)	1.24 (1.13–1.35)	0.74 (0.58–0.90)	2.75 (2.37–3.07)	2.47 (2.01–3.03)
Vietnam	0.62 (0.58–0.66)	2.34 (2.06–2.62)	1.72 (1.41–2.03)	6.44 (5.84–6.94)	3.80 (3.17–4.49)
Myanmar	0.42 (0.37–0.47)	1.37 (1.31–1.43)	0.95 (0.84–1.06)	3.55 (3.47–3.61)	3.29 (2.83–3.87)
Philippines	0.31 (0.27–0.35)	1.11 (0.95–1.27)	0.80 (0.60–1.00)	2.98 (2.48–3.40)	3.59 (2.73–4.73)
Nigeria	0.01 (0.01–0.01)	0.32 (0.29–0.35)	0.31 (0.28–0.34)	1.16 (0.95–1.41)	49.70 (40.87–67.49)
Cambodia	0.12 (0.08–0.16)	0.31 (0.30–0.32)	0.18 (0.13–0.23)	0.68 (0.56–0.78)	2.50 (1.82–3.92)
Pakistan	0.19 (0.15–0.23)	1.24 (1.18–1.30)	1.05 (1.03–1.07)	3.93 (3.52–4.44)	6.66 (5.78–8.02)
Brazil	0.42 (0.38–0.46)	0.66 (0.38–0.94)	0.24 (0.01–0.47)	0.88 (0.02–1.58)	1.56 (1.02–2.00)
Guinea	0.02 (0.02–0.02)	0.32 (0.30–0.34)	0.30 (0.28–0.32)	1.12 (0.95–1.33)	15.62 (15.47–15.79)
Japan	0.58 (0.32–0.84)	0.37 (0.21–0.53)	−0.21 (−0.32–−0.10)	−0.77 (−1.28–−0.34)	0.64 (0.63–0.67)
Nepal	0.10 (0.06–0.14)	0.28 (0.20–0.36)	0.17 (0.12–0.22)	0.64 (0.42–0.90)	2.64 (2.57–2.79)
Other	1.33 (1.22–1.44)	5.37 (4.63–6.11)	4.04 (3.18–4.90)	15.10 (13.18–16.66)	4.02 (3.20–5.02)

Table S3. Trends in CH_4 emission intensity from rice paddies: Global and top 15 country-level analysis (1961–2020).

Country	Overall trend (g CH_4/kg rice/decade)	Increase (%Share by grid)	Decrease (%Share by grid)	Increase (%Share by area)	Decrease (%Share by area)
Global	-0.78*	32.7	67.3	28.7	71.3
India	-2.24*	12.7	87.3	18.0	82.0
China	-0.36*	59.8	40.2	67.0	33.0
Bangladesh	-2.44*	0.4	99.6	0.0	100.0
Indonesia	-0.17‡	25.0	75.0	45.6	54.4
Thailand	-1.74*	6.6	93.4	6.3	93.7
Vietnam	-2.84*	6.2	93.8	11.1	88.9
Myanmar	-2.02*	0.6	99.4	0.5	99.5
Philippines	-3.86*	0.5	99.5	0.6	99.4
Nigeria	-0.78†	14.4	85.6	21.3	78.7
Cambodia	-3.98*	0.2	99.8	0.1	99.9
Pakistan	0.80‡	52.1	47.9	65.4	34.6
Brazil	-2.82*	2.9	97.1	2.2	97.8
Guinea	3.39*	97.7	2.3	97.9	2.1
Japan	0.05‡	67.6	32.4	62.9	37.1
Nepal	-0.26‡	44.2	55.8	45.6	54.4

Note:

"Overall trend" refers to the linear trend in annual total CH_4 emission intensity (total emissions divided by total rice production) over the past 60 years. * indicates $p < 0.001$; † indicates $p < 0.1$; ‡ indicates results that are not statistically significant. The proportion statistics are based on slope values calculated individually for each grid cell.

Table S4. Summary of data used in this study.

Category	Data source	Original resolution	Used in CH4MOD	Used in CH4-ML	Used in CH4-EF
Temperature*	ERA5[3]	0.25°, Daily	√	√	-
Soil Sand Fraction	HWSD[4]	30″	√	√	-
Rice Harvested Area	Xie et al.[5]	5′, Annual	√	√	√
Rice Yield	M3[11]<br>FAOSTAT[7]	5′, Year 2000<br>Country, Annual	√	√	√
Crop Rotation	Waha et al.[8]	0.5°	√	√	√
Crop Phenology	IRRI RiceAtlas[9]	Province	√	√	√
Manure Addition	Zhang et al.[10]	5′, Annual	√	√	√
Straw Incorporation†	This study[43]	Province / Country	√	√	√
Water Management‡	This study[43]	Province / Country	√	√	√

Note:

* CH4MOD used daily temperature, CH4-ML used accumulated growing-season temperature.

† A common feature of the three approaches is the use of the same straw incorporation rate, with the amount of straw estimated based on rice yield. However, they differ in the treatment of organic matter inputs. The CH4-EF method considers only the amount of straw returned to the field after harvest. In contrast, both CH4-ML and CH4MOD additionally account for the remaining belowground biomass, such as roots and stubble from the previous season. Furthermore, CH4MOD includes a dedicated module that estimates root exudates based on aboveground biomass, which are also considered as methanogenic substrates.

‡ CH4MOD and CH4-ML considered 8 water regimes, while CH4-EF combined them into 4 categories. Including: CF (F), SD (FDF), MD (FDFM, FDM, FM, M), Rainfed (RFW, RFD). see Methods for details.

Table S5. Performance of machine-learning models for CH_4 flux prediction under random splitting, repeated random splitting, and site-independent cross-validation.

	Random Forest	XGBoost	Extra Trees
Random test R^2	0.80	0.81	0.77
Random test RMSE (g m^{-2})	10.07	9.48	10.66
Random test MAE (g m^{-2})	5.71	5.75	6.12
Repeated random R^2	0.86 ± 0.03	0.86 ± 0.03	0.83 ± 0.04
Repeated random RMSE (g m^{-2})	8.84 ± 0.85	8.77 ± 0.80	9.64 ± 0.97
Repeated random MAE (g m^{-2})	5.61 ± 0.46	5.74 ± 0.46	6.05 ± 0.51
Site-independent R^2	0.67 ± 0.16	0.63 ± 0.23	0.63 ± 0.15
Site-independent RMSE (g m^{-2})	12.54 ± 2.10	13.04 ± 2.77	13.47 ± 2.33
Site-independent MAE (g m^{-2})	8.53 ± 1.50	9.14 ± 1.85	8.95 ± 1.28

Note:

(1) The random test set was based on one random 80/20 train–test split.

(2) Repeated random validation was based on 100 repeated random 80/20 train–test splits.

(3) Site-independent cross-validation was conducted using five-fold GroupKFold by geographical site, in which observations from the same site were assigned to the same fold.

(4) Values are reported as mean ± standard deviation for repeated random validation and site-independent cross-validation.

(5) Random Forest was selected for global prediction and is denoted as CH4-ML in this study, because it showed the best overall balance between predictive performance and stability under site-independent cross-validation, while avoiding negative predictions occasionally generated by XGBoost.

Table S6. Published estimates from existing studies.

Study/dataset	Method type	Period	Comparison domain	Reference
Chen et al. (2025), GRPI	High-resolution bottom-up rice-specific inventory combining Landsat-derived inundation/rice maps with rice-specific emission factors	2022	Global; selected countries	[44]
Lu et al. (2021)	Top-down Bayesian atmospheric inversion using GOSAT and in situ CH_4 observations	2010–2017	Global	[45]
Zhang et al. (2026)	Multi-method rice-paddy GHG estimate combining a data-driven model, a process-based ecosystem model, and field-experiment meta-analysis	2001–2020	Global; selected countries	[46]
CEDS_v2024	Harmonized bottom-up global sectoral inventory based on country/sector activity data, emission factors, and inventory scaling	1970–2022	Global; selected countries	[47]
EDGAR_2025	Global bottom-up IPCC-based gridded inventory using activity data and emission factors, with updated rice-cultivation CH_4 emission factors	1970–2024	Global; selected countries	[32]
FAOSTAT_v2025	FAOSTAT/IPCC Tier 1 rice-cultivation CH_4 estimate using harvested rice area, regional management assumptions, and default emission factors	1961–2023	Global; selected countries	[7]
Chen et al. (2024)	Atmospheric-observation-informed attribution supported by bottom-up rice CH_4 estimates based on rice expansion, cultivation conditions, and emission factors	2008–2018	Africa	[48]
Zhou et al. (2024)	Field-observation-based machine-learning upscaling of paddy CH_4 emissions across Monsoon Asia using environmental and management drivers	2001–2020	Monsoon Asia	[49]

Ouyang et al. (2023)	Machine-learning upscaling using eddy-covariance CH_4 fluxes and MODIS remote-sensing data to produce 5-km gridded estimates across Monsoon Asia	2001–2015	Monsoon Asia; selected countries	[50]
REAS_v2.1	Regional bottom-up Asian emission inventory using country/sector activity data and emission parameters, with monthly 0.25° gridding	2000–2008	Asian countries	[51]
Li et al. (2025)	Historical China rice CH_4 inventory based on integrated field measurements, process-based modeling, machine learning, and water-saving irrigation data	1949–2021	China	[52]
Li et al. (2020)	Improved IPCC emission-factor inventory based on provincial agricultural activity data and management-specific scaling factors	1980–2014	China	[53]
PKU_CH4_v2	Gridded bottom-up anthropogenic CH_4 inventory for China using sectoral activity data, emission factors, and spatial proxy downscaling	2000–2019	China	[54]
Guo et al. (2025), CHN-CH4	Bottom-up gridded anthropogenic CH_4 inventory for China; rice-cultivation emissions estimated from rice area, provincial emission factors, growing period, and remote-sensing rice maps	1990–2020	China	[55]
Zhang et al. (2022)	Top-down atmospheric inversion using satellite and surface CH_4 observations, with subnational and sectoral attribution	2010–2017	China	[56]
Sun et al. (2020)	Regional empirical/statistical model trained with 835 field measurements and driven by high-resolution spatial data	2015	China	[57]
Yan et al. (2003a)	Measurement-synthesis emission-factor estimate using 204 season-treatment observations, management factors, and spatial rice-area data	1995	China	[58]
Chen et al. (2013)	Literature-synthesis re-estimation of Chinese CH_4 emissions from rice paddies based on reviewed measurements and reports	2008	China	[59]
Wang et al. (2021)	Process-based DNDC model simulation for Chinese rice CH_4 emissions	2012	China	[60]

Xia et al. (2024)	Optimized IPCC 2019 emission-factor method using high-resolution rice distribution and updated daily emission factors	2015	China	[61]
Bhatia et al. (2013)	State-wise IPCC-based emission-factor inventory using rice ecosystem activity data and emission coefficients	1980–2007	India	[62]
Bhatia et al. (2012)	InfoCrop process-model simulation with spatial upscaling for rice–wheat systems in India	2005	India	[63]
Nene et al. (1990)	Early field-measurement-based estimate using chamber field CH_4 flux observations and paddy-area extrapolation	1988	India	[64]
Cao et al. (1996)	Process-based methane emission model applied globally using rice productivity, soil organic matter, and spatial seasonal rice-paddy drivers	1993	India	[65]
Gupta et al. (2009)	NATCOM state-wise emission-factor inventory using water-regime-specific Indian emission factors and rice-area activity data	1979–2006	India	[66]
Sahil et al. (2024)	Synthesis of 726 field measurements to derive district-level water-regime-specific emission factors and assess mitigation options for Indian rice fields	1966–2017	India	[67]
Yan et al. (2003b)	Region-specific emission-factor estimate based on assembled Asian field measurements stratified by climate, soil, water regime, and organic input	1995	India	[68]
Pathak et al. (2005)	Calibrated DNDC process-model simulation with spatial upscaling for Indian rice fields	2000	India	[69]
Parashar et al. (1996)	National methane campaign based on chamber field measurements across Indian rice regions and paddy-area extrapolation	1991	India	[70]

Table S7. Fixed-driver decomposition and residual term for rice CH_4 emission changes over 1961–2020. Values indicate changes in annual CH_4 emissions between 1961 and 2020 and are expressed in Tg CH_4 yr^{-1}. Δbaseline is the total emission change in the reference simulation. Apparent contributions were estimated from fixed-driver simulations in CH4MOD.

Region	Δbaseline	Temperature	Harvested area	Root exudates	Straw incorporation	Farmyard manure	Residual
Global	28.67	2.31	10.92	11.68	6.87	0.82	−3.93
India	6.77	0.63	2.19	2.82	1.69	0.21	−0.77
China	5.39	0.31	0.80	1.58	2.49	0.18	0.03
Bangladesh	1.21	0.05	0.43	0.51	0.44	0.03	−0.25
Indonesia	3.25	0.24	1.47	1.41	0.54	0.14	−0.55
Thailand	0.89	0.10	0.54	0.29	0.20	0.00	−0.24
Vietnam	1.99	0.13	0.88	0.99	0.57	0.05	−0.63
Myanmar	1.02	0.09	0.50	0.45	0.25	0.02	−0.29
Philippines	0.93	0.09	0.39	0.44	0.28	0.02	−0.29
Nigeria	0.28	0.02	0.28	0.09	0.00	0.00	−0.11
Cambodia	0.22	0.02	0.07	0.10	0.06	0.00	−0.03
Pakistan	1.09	0.06	0.79	0.32	0.00	0.04	−0.12
Brazil	0.43	0.03	−0.82	0.55	0.00	0.01	0.66
Guinea	0.30	0.02	0.30	−0.03	0.00	0.01	0.00
Japan	−0.08	0.02	−0.28	0.01	0.05	0.00	0.12
Nepal	0.16	0.00	0.06	0.04	0.00	0.02	0.04

Table S8 Management sensitivity scenarios for evaluating the effects of water management and straw incorporation assumptions on CH4MOD-estimated rice CH_4 emissions.

Scenario	1961-1980	1981-2000	2001-2020
BASE	Baseline	Baseline	Baseline
WAT_10	+10% F	Baseline	+10% M
STR_10	−10% straw	Baseline	+10% straw
COM_10	+10% F & −10% straw	Baseline	+10% M & +10% straw
WAT_20	+20% F	Baseline	+20% M
STR_20	−20% straw	Baseline	+20% straw
COM_20	+20% F & −20% straw	Baseline	+20% M & +20% straw
WAT_30	+30% F	Baseline	+30% M
STR_30	−30% straw	Baseline	+30% straw
COM_30	+30% F & −30% straw	Baseline	+30% M & +30% straw

Table S9. Summary of global CH_4 emissions under CH4MOD management sensitivity scenarios.

Scenario	1961 emissions (Tg CH_4 yr^{-1})	2020 emissions (Tg CH_4 yr^{-1})	Growth factor (1961–2020)	Difference from BASE in 2020
BASE	10.13	39.53	3.90	—
WAT_10	11.20	37.52	3.35	−5.1%
STR_10	9.67	41.30	4.27	+4.5%
COM_10	10.72	39.19	3.66	−0.9%
WAT_20	12.27	35.50	2.89	−10.2%
STR_20	9.17	43.06	4.70	+8.9%
COM_20	11.21	38.65	3.45	−2.2%
WAT_30	13.33	33.48	2.51	−15.3%
STR_30	8.65	44.82	5.18	+13.4%
COM_30	11.65	37.92	3.26	−4.1%

Supplementary References

1. Hu Q, Li J and Xie H et al. Global methane emissions from rice paddies: CH4MOD model development and application. iScience 2024; 27: 111237.
2. Huang Y, Sass RL and Fisher FM. A semi-empirical model of methane emission from flooded rice paddy soils. Glob Change Biol 1998; 4: 247–68.
3. Soci C, Hersbach H and Simmons A et al. The ERA5 global reanalysis from 1940 to 2022. Q J R Meteorol Soc 2024; 150: 4014–48.
4. FAO and IIASA. Harmonized World Soil Database Version 2.0. Rome and Laxenburg: FAO and IIASA; 2023.
5. Xie H, Li J and Li T et al. GloRice, a global rice database (v1.0): I. Gridded paddy rice annual distribution from 1961 to 2021. Sci Data 2025; 12: 182.
6. Monfreda C, Ramankutty N and Foley JA. Farming the planet: 2. Geographic distribution of crop areas, yields, physiological types, and net primary production in the year 2000. Glob Biogeochem Cycles 2008; 22: 2007GB002947.
7. FAO. FAOSTAT: Statistical Database. Food and Agriculture Organization of the United Nations, Rome, Italy. https://www.fao.org/faostat/ (2024).
8. Waha K, Dietrich JP and Portmann FT et al. Multiple cropping systems of the world and the potential for increasing cropping intensity. Glob Environ Change 2020; 64: 102131.
9. Laborte AG, Gutierrez MA and Balanza JG et al. RiceAtlas, a spatial database of global rice calendars and production. Sci Data 2017; 4: 170074.
10. Zhang B, Tian H and Lu C et al. Global manure nitrogen production and application in cropland during 1860–2014: a 5 arcmin gridded global dataset for Earth system modeling. Earth Syst Sci Data 2017; 9: 667–78.
11. Ramankutty N, Evan AT and Monfreda C et al. Farming the planet: 1. Geographic distribution of global agricultural lands in the year 2000. Glob Biogeochem Cycles 2008; 22: 2007GB002952.
12. Akratos CS, Tekerlekopoulou AG and Vasiliadou IA et al. Cocomposting of olive mill waste for the production of soil amendments. In: Olive Mill Waste. Amsterdam: Elsevier; 2017, 161–82.
13. He P. China Organic Fertilizer Nutrient Records. Beijing: China Agriculture Press; 1999.
14. Han PF. Global changes in soil organic carbon in croplands. PhD Thesis. Beijing: University of Chinese Academy of Sciences; 2016.
15. Ministry of Ecology and Environment of the People’s Republic of China (MEE). The People’s Republic of China’s Third National Communication on Climate

Change. 2018; https://www.mee.gov.cn/ywgz/ydqhbh/wsqtkz/201907/ P020190701762678052438.pdf.

16. ESCAP. Status of straw management in Asia-Pacific and options for integrated straw management. 2018; https://hdl.handle.net/20.500.12870/4468.
17. IPCC. Revised 1996 IPCC Guidelines for National Greenhouse Gas Inventories. 1996.
18. Asian Development Bank and United Nations Development Programme. Asia Least Cost Greenhouse Gas Abatement Strategy (ALGAS). 1998.
19. IPCC. 2006 IPCC Guidelines for National Greenhouse Gas Inventories. 2006.
20. Huang Y. Modeling methane emission from rice paddies with various agricultural practices. J Geophys Res 2004; 109: D08113.
21. Xie B, Zhou Z and Zheng X et al. Modeling methane emissions from paddy rice fields under elevated atmospheric carbon dioxide conditions. Adv Atmos Sci 2010; 27: 100–14.
22. Huang Y, Sass RL and Fisher FM. Methane emission from Texas rice paddy soils. 1. Quantitative multi-year dependence of CH4 emission on soil, cultivar and grain yield. Glob Change Biol 1997; 3: 479–89.
23. Huang Y, Sass RL and Fisher FM. Methane emission from Texas rice paddy soils. 2. Seasonal contribution of rice biomass production to CH4 emission. Glob Change Biol 1997; 3: 491–500.
24. Lundberg SM, Erion G and Chen H et al. Explainable AI for Trees: From Local Explanations to Global Understanding. arXiv:1905.04610; 2019.
25. Lundberg SM, Erion GG and Lee SI. Consistent Individualized Feature Attribution for Tree Ensembles. arXiv:1802.03888; 2018.
26. Bergstra J and Bengio Y. Random search for hyper-parameter optimization. J Mach Learn Res 2012; 13: 281–305.
27. Kohavi R. A study of cross-validation and bootstrap for accuracy estimation and model selection. Proc IJCAI 1995; 14: 1137–45.
28. Roberts, D. R., Bahn, V., Ciuti, S., et al. Cross-validation strategies for data with temporal, spatial, hierarchical, or phylogenetic structure. Ecography 2017, 40, 913–929.
29. Meyer, H., & Pebesma, E. Predicting into unknown space? Estimating the area of applicability of spatial prediction models. Methods in Ecology and Evolution 2021, 12, 1620–1633.
30. IPCC. 2019 Refinement to the 2006 IPCC Guidelines for National Greenhouse Gas Inventories. 2019.

31. Saunois M, Martinez A and Poulter B et al. Global Methane Budget 2000–2020. Earth Syst Sci Data 2025; 17: 1873–958.

32. Crippa M et al. GHG Emissions of All World Countries. European Commission. Joint Research Centre. & IEA; 2024. https://data.jrc.ec.europa.eu/collection/EDGAR.

33. US EPA. Data Annex: Global Non-CO_2 Greenhouse Gas Emissions Projections. U.S. Environmental Protection Agency, Washington, D.C., USA; 2012. https://www.epa.gov/global-mitigation-non-co2-greenhouse-gases.

34. Höglund-Isaksson L, Gómez-Sanabria A and Klimont Z et al. Technical potentials and costs for reducing global anthropogenic methane emissions in the 2050 timeframe –results from the GAINS model. Environ Res Commun 2020; 2: 025004.

35. Wang J, Ciais P and Smith P et al. The role of rice cultivation in changes in atmospheric methane concentration and the Global Methane Pledge. Glob Change Biol 2023; 29: 2776–89.

36. Peng S et al. Inventory of anthropogenic methane emissions in mainland China from 1980 to 2010. Atmos Chem Phys 2016; 16: 14545–62.

37. Bouman BAM. Water management in irrigated rice: coping with water scarcity. Los Baños: Int Rice Res Inst; 2007.

38. Huke RE and Huke EH. Rice area by type of culture: south, southeast and east Asia: a revised and updated database. Manila: Int Rice Res Inst; 1997.

39. GRiSP. Rice almanac, 4th edn. Los Baños: Int Rice Res Inst; 2013.

40. Wang J, Akiyama H and Yagi K et al. Controlling variables and emission factors of methane from global rice fields. Atmos Chem Phys 2018; 18: 10419–31.

41. Yan X, Yagi K and Akiyama H et al. Statistical analysis of the major variables controlling methane emission from rice fields. Glob Change Biol 2005; 11: 1131–41.

42. Yan X, Akiyama H and Yagi K et al. Global estimations of the inventory and mitigation potential of methane emissions from rice cultivation conducted using the 2006 IPCC Guidelines. Glob Biogeochem Cycles 2009; 23: GB2002.

43. This study, see Methods for details.

44. Chen Z, Lin H and Balasus N et al. Global Rice Paddy Inventory (GRPI): a high-resolution inventory of methane emissions from rice agriculture based on Landsat satellite inundation data. Earths Future 2025; 13: e2024EF005479.

45. Lu X, Jacob DJ and Zhang Y et al. Global methane budget and trend, 2010–2017: complementarity of inverse analyses using in situ (GLOBALVIEWplus CH4 ObsPack) and satellite (GOSAT) observations. Atmos Chem Phys 2021; 21: 4637–57.

46. Zhang J, Tian H and Liang X-Z et al. Global rice paddy greenhouse gas emissions have doubled over the past six decades driven by area expansion and intensified residue incorporation. Nat Food 2026; 7:1-14.

47. Hoesly R and Smith S. CEDS v_2024_04_01 Release Emission Data. Zenodo 2024; doi:10.5281/zenodo.10904361.

48. Chen Z, Balasus N and Lin H et al. African rice cultivation linked to rising methane. Nat Clim Change 2024; 14: 148–51.

49. Zhou H, Tao F and Chen Y et al. Paddy rice methane emissions, controlling factors, and mitigation potentials across Monsoon Asia. Sci Total Environ 2024; 935: 173441.

50. Ouyang Z, Jackson RB and McNicol G et al. Paddy rice methane emissions across Monsoon Asia. Remote Sens Environ 2023; 284: 113335.

51. Kurokawa J, Ohara T and Morikawa T et al. Emissions of air pollutants and greenhouse gases over Asian regions during 2000–2008: Regional Emission inventory in ASia (REAS) version 2. Atmos Chem Phys 2013; 13: 11019–58.

52. Li Y, Zhang L and Li H et al. Co-benefits of water-saving irrigation for mitigating CH4 emissions from rice cultivation in China: insights from the past seven decades. Environ Sci Technol 2025; 59: 23949–61.

53. Li N, Shang L and Yu Z et al. Estimation of agricultural greenhouse gases emission in interprovincial regions of China during 1996–2014. Nat Hazards 2020; 100: 1037–58.

54. Liu L, Peng S and Wu L et al. PKU-CH4_v2: gridded anthropogenic methane emissions in China during 2000–2019. Figshare 2021; doi:10.6084/m9.figshare.15151353.

55. Guo F, Dai F and Gong P et al. CHN-CH4: a gridded (0.1° × 0.1°) anthropogenic methane emission inventory of China from 1990 to 2020. Earth Syst Sci Data 2025; 17: 4799–819.

56. Zhang Y, Fang S and Chen J et al. Observed changes in China's methane emissions linked to policy drivers. Proc Natl Acad Sci USA 2022; 119: e2202742119.

57. Sun J, Zhang X and Wan L et al. Re-estimating methane emissions from Chinese paddy fields based on a regional empirical model and high-resolution spatial data. Environ Pollut 2020; 265: 115017.

58. Yan X, Cai Z and Ohara T et al. Methane emission from rice fields in mainland China: amount and seasonal and spatial distribution. J Geophys Res Atmos 2003a; 108: 4505.

59. Chen H, Zhu Q and Peng C et al. Methane emissions from rice paddies, natural wetlands, and lakes in China: synthesis and new estimate. Glob Change Biol 2013; 19: 19–32.

60. Wang W, Zeng W and Chen D et al. Mapping rice paddy methane emissions in China using the DNDC model. Agric For Meteorol 2021; 307: 108368.

61. Xia W, Fan C and Zhang G. Estimation of methane emissions from Chinese rice paddies based on an optimized IPCC method. Acta Ecol Sin 2024; 44: 9826–35. (in Chinese)

62. Bhatia A, Jain N and Pathak H. Methane and nitrous oxide emissions from Indian rice paddies, agricultural soils and crop residue burning. Greenhouse Gases Sci Technol 2013; 3: 196–211.

63. Bhatia A, Aggarwal PK and Jain N et al. Greenhouse gas emission from rice- and wheat-growing areas in India: spatial analysis and upscaling. Greenhouse Gases Sci Technol 2012; 2: 115–25.

64. Neue HU, Becker-Heidmann P and Scharpenseel HW. Organic matter dynamics, soil properties, and cultural practices in rice lands and their relationship to methane production. In: Bouwman AF (ed.). Soils and the Greenhouse Effect. Chichester: John Wiley & Sons, 1990, 457–66.

65. Cao M, Gregson K and Marshall S et al. Global methane emissions from rice paddies. Chemosphere 1996; 33: 879–97.

66. Gupta PK, Gupta V and Sharma C et al. Development of methane emission factors for Indian paddy fields and estimation of national methane budget. Chemosphere 2009; 74: 590–8.

67. Sahil FM, Nayak AK and Pathak H et al. Long-term changes in methane emissions from Indian rice cultivation. Glob Biogeochem Cycles 2024; 38: e2024GB008107.

68. Yan X, Ohara T and Akimoto H. Development of region-specific emission factors and estimation of methane emission from rice fields in the East, Southeast and South Asian countries. Glob Change Biol 2003b; 9: 237–54.

69. Pathak H, Li C and Wassmann R. Greenhouse gas emissions from Indian rice fields: calibration and upscaling using the DNDC model. Biogeosciences 2005; 2: 113–23.

70. Parashar DC, Mitra AP and Gupta PK et al. Methane budget from paddy fields in India. Chemosphere 1996; 33: 737–57.
